# A multisource clinical deep learning model for early identification of mental disorders in first-visit psychiatric patients

**DOI:** 10.3389/fpsyt.2026.1988768

**Published:** 2026-10-28

**Authors:** Yu Zhang, Zhan Gao, Wenyu Ge, Zhiyu Cao, Hongyan Zhu, Yuchao Ma, Weiwei Wang, Yuqing Song, Yu Li, Xiangna Zheng

**Affiliations:** 1 Department of Psychiatry, Heilongjiang Provincial Hospital, Harbin, China; 2 Department of General Practice, Heilongjiang Provincial Hospital, Harbin, China; 3 Department of Dentistry, Heilongjiang Provincial Hospital, Harbin, China; 4 Department of Neurology, Heilongjiang Provincial Hospital, Harbin, China; 5 Outpatient Comprehensive Clinic, Heilongjiang Provincial Hospital, Harbin, China; 6 Discipline Inspection and Supervision Office, Heilongjiang Provincial Hospital, Harbin, China

**Keywords:** clinical validation, deep learning, first-visit psychiatric patients, mental disorders, multi-label classification, psychiatric diagnosis, SCL-90

## Abstract

**Introduction:**

Mental disorders often present with overlapping symptoms at the first psychiatric visit, making early identification difficult. This study included 5802 first-visit psychiatric patients and developed a TUCH-FT for multi-label classification of mental disorders using demographic information, clinical history, and item-level Symptom Checklist-90 data. Final diagnostic labels were determined through longitudinal follow-up, treatment response, and psychiatric expert evaluation. The original FT-Transformer places heterogeneous clinical variables in a single sequence, which may weaken source-specific structures, inadequately represent unclear clinical histories, and fail to model directional cross-source interactions and hierarchical comorbidity relationships.

**Methods:**

To overcome these limitations, this study proposes a three-branch clinical feature encoding method, an uncertainty-aware embedding method, a cross-group multi-head attention fusion mechanism, and a hierarchical multi-label classification method to address the challenges of heterogeneous clinical information, ambiguous clinical histories, the interaction of information from multiple sources, and the modelling of comorbidity relationships.

**Results:**

TUCH-FT achieved a Macro-F1 of 0.803, Micro-F1 of 0.842, Macro-average precision of 0.838, Hamming loss of 0.095, and label ranking average precision of 0.876. In a temporally separated, same-centre validation cohort of 1, 272 patients, TUCH-FT demonstrated good calibration performance, with the lowest Brier score observed for psychotic disorders (0.072) and the highest for anxiety disorders (0.134), while expected calibration errors ranged from 0.031 to 0.067.

**Discussion:**

Although the exact-match accuracy for complete diagnostic combinations was 60.8%, indicating some limitations in predicting co-occurring disorders, TUCH-FT may still provide a useful reference for differential assessment among first-visit psychiatric patients presenting with mood-related symptoms.

## 1 Introduction

Mental disorders are characterised by complex symptoms, significant overlap between conditions, and difficulties in early diagnosis (1). Depressive disorders, bipolar disorder, anxiety disorders, obsessive-compulsive disorder and psychotic disorders often present with similar emotional, cognitive and physical symptoms at the time of the initial consultation; relying solely on a single clinical interview or scale scores can easily lead to missed diagnoses, misdiagnoses or delayed diagnosis (2, 3). This is particularly true for first-time patients who have not previously received systematic psychiatric treatment; their clinical history information (CHI) is often incomplete, and symptoms such as mania, hypomania, psychotic symptoms and a history of previous episodes may be difficult to confirm accurately due to recall bias, poor insight or a lack of corroborating evidence from family members (4). Clinicians typically need to narrow down the diagnostic range through multiple rounds of interviews, scale assessments, therapeutic observations and follow-up, which increases the utilisation of healthcare resources and imposes a financial burden on patients due to unnecessary tests and repeat visits (5). Therefore, conducting auxiliary identification of the likely nature of the disorder at the initial consultation stage can provide a basis for targeted questioning and differential diagnosis, thereby improving the efficiency of diagnosis and treatment, and is of significant clinical importance.

The Symptom Checklist-90 (SCL-90) is capable of reflecting patients’ recent psychological symptoms and their severity across multiple dimensions, including somatisation, obsessive-compulsive symptoms, interpersonal sensitivity, depression, anxiety, hostility, phobias, paranoia and psychosis, and has been widely used in the screening of mental and psychological states (6–10). Gomez et al. validated the construct validity of the SCL-90-R scale; using various factor analysis methods to assess its underlying structure in a sample of 2, 090 adolescents, the results showed that a model comprising nine symptom dimensions provided the best fit. Furthermore, several symptom dimensions were found to be associated with external family functioning indicators, demonstrating that the SCL-90-R possesses good construct validity and the capacity to assess psychological symptoms (11).

However, the SCL-90 is a self-report instrument designed to assess the severity and breadth of psychological symptoms rather than to establish a standalone psychiatric diagnosis. Discordance has also been reported between SCL-90 scores and clinician-rated psychopathology, indicating that its results should be interpreted together with clinical interviews, medical history and comprehensive clinical assessment (12, 13). Therefore, the total score, factor scores and screening thresholds of the SCL-90 should not be directly equated with clinical diagnostic categories.

In recent years, machine learning methods have provided new technical tools for the analysis of complex clinical tabular data. Alves et al. utilised EMR clinical records to construct a machine learning model to predict PHQ-9 scores in patients with MDD; the model achieved an AUC of 0.81, a PPV of 0.71 and an NPV of 0.76 (14). Terhorst et al. employed machine learning models to identify major depressive episodes (MDE) using the PHQ-9, QIDS-16 and HAM-D-17 scales; the QIDS-16 model demonstrated the best performance, with an AUC of 0.94, representing a 14% improvement in F1 score and an 8% increase in balanced accuracy compared to the traditional total score threshold method (15). Kim et al. conducted a diagnostic meta-analysis based on machine learning methods to address the issue of insufficient accuracy in PHQ-9 depression screening on mobile devices. The results showed that the pooled sensitivity and specificity of the models reached 0.797 and 0.850, respectively (16). These studies addressed different prediction targets and used different datasets and evaluation measures; their reported performance values therefore provide background context rather than a direct benchmark for TUCH-FT.

Psychiatric diagnoses are not mutually exclusive, and a single patient may simultaneously meet the criteria for multiple disorders. Accordingly, clinical diagnosis prediction can be formulated as a multi-label learning problem in which multiple diagnostic labels are predicted for each patient while accounting for dependencies among co-occurring diagnoses. Previous clinical multi-label models have addressed related but distinct prediction tasks. Doctor AI used a recurrent neural network to predict diagnosis and medication categories at subsequent visits from longitudinal electronic health records (17). Zhang et al. used a convolutional encoder and residual network to predict correlated diagnoses from clinical notes (18). These studies establish the value of multi-label prediction, but their inputs and prediction settings differ from the present task, which uses demographic information, clinical history and item-level SCL-90 responses available at the first psychiatric visit.

In mental disorder detection specifically, the dual-branch network (DBN) proposed by Zhang, Cui and Peng (1) uses resting-state quantitative electroencephalogram features, an attention mechanism and SHAP analysis. Its EEG-based inputs and classification setting differ from the first-visit clinical data and simultaneous multi-label predictions considered in this study.

For heterogeneous tabular data, Transformer-based architectures can convert numerical and categorical variables into feature-token representations and use self-attention to model interactions among variables. The FT-Transformer has demonstrated competitive performance across multiple tabular learning tasks, although no modelling approach is universally superior across all datasets (19). Therefore, this study compared TUCH-FT with linear, tree-based and neural-network baselines rather than assuming the superiority of deep learning.

The original FT-Transformer typically places all variables within the same feature sequence, without explicitly distinguishing between the three categories of information—demographic information (DI), CHI and SCL-90 symptoms—in terms of their clinical significance and information structure; this may weaken the model’s ability to represent features from different information sources. Furthermore, due to recall bias, insufficient insight or limited understanding of the illness, information on past symptoms in first-time patients is often ‘unclear’; yet the original model struggles to distinguish between missing data and uncertainty, and is therefore unable to make full use of such CHI. Furthermore, mental disorders are characterised by significant symptom overlap and comorbidity; there are complex interactions between different sources of CHI, and there are hierarchical relationships between disease categories. In this study, the five diagnostic labels are organised under three broad categories: affective disorders (depressive and bipolar disorders), anxiety-related disorders (anxiety and obsessive-compulsive disorders), and psychotic disorders.Traditional single-sequence modelling approaches struggle to effectively capture the interactions between multi-source information and the hierarchical structure of diseases.

Consequently, given the characteristics of patients attending their first psychiatric consultation—such as the heterogeneous nature of CHI from multiple sources, the prevalence of uncertain medical histories, and the overlap of symptoms and comorbidity in disease diagnosis—there is an urgent need to develop an auxiliary identification method capable of simultaneously preserving the characteristics of different CHI sources, utilising uncertain information, and modelling cross-source interactions. This study utilises the FT-Transformer as its foundational framework and introduces a hierarchical cross-attention mechanism tailored to the clinical characteristics of mental disorders, thereby constructing the TUCH-FT to enhance multi-label recognition capabilities for mental disorders at the initial consultation stage. The main work and contributions of this study are as follows:

1. A longitudinal clinical cohort of patients attending their first psychiatric consultation was established. DI, CHI and item-level symptom profiles from the SCL-90 were collected; the final diagnosis was determined through a combination of follow-up assessments and comprehensive expert judgement, thereby providing clinical labels for model training.
2. To address the issue of insufficient feature representation caused by structural differences in multi-source CHI, a three-branch clinical feature encoding (TCFE) method is proposed. Independent encoding is performed according to the sources of DI, CHI and SCL-90 symptoms, whilst preserving the feature correlations between different information groups to enhance the representational capacity for heterogeneous data.
3. To address the difficulty in utilising ‘unclear’ information in initial medical histories, an uncertainty-aware embedding (UE) module is proposed. By jointly modelling the medical history status and the degree of information clarity, the method distinguishes between ‘none’ and ‘unclear’ states, thereby preserving the latent value of clinically uncertain information.
4. To better integrate demographic information, medical history and symptom profiles, we developed a cross-group multi-head attention fusion (CMAF) mechanism.
5. To address the difficulty in representing hierarchical relationships and comorbidity characteristics in mental disorders, a hierarchical multi-label classification (HMC) structure is constructed. By jointly predicting disease categories and the probabilities of specific diseases, the model’s ability to recognise complex mental disorder states is improved.
6. The TUCH-FT model is established, and its effectiveness is validated through comparative experiments, ablation studies and independent clinical data.

## 2 Materials and methods

### 2.1 Participants

This study employed a prospective observational cohort design and enrolled 5802 patients. Patient recruitment and longitudinal follow-up were conducted at Heilongjiang Provincial Hospital between February and December 2023 under ethics approval no. [2023] 003, granted in January 2023. The secondary analysis of the collected data and model development and validation were separately approved in 2026 under approval no. [2026] 055.

Following longitudinal follow-up, assessment of treatment response and comprehensive evaluation by psychiatric specialists, 3243 patients were diagnosed with at least one target mental disorder, whereas the remaining 2559 patients (44.1%) did not meet the diagnostic criteria for any target mental disorder. All patients were retained in the analysis. Diagnostic outcomes were encoded as five-dimensional multi-label vectors in the fixed order of depression, bipolar disorder, anxiety disorder, obsessive-compulsive disorder and psychotic disorder. For each disease, a value of 1 indicated a positive diagnosis and a value of 0 indicated a negative diagnosis. Therefore, patients without any of the five target mental disorders were encoded as [0, 0, 0, 0, 0], representing negative diagnoses for depression, bipolar disorder, anxiety disorder, obsessive-compulsive disorder and psychotic disorder, respectively. Each patient was assigned a unique study code, and direct personal identifiers were stored separately from the analytical dataset. Only de-identified data were used for model development and evaluation.

The inclusion criteria are as follows: aged between 18 and 55 years; presenting for the first time at a psychiatric clinic due to symptoms of depression, mood swings or other mood-related disorders; no previous definitive diagnosis or systematic treatment at a specialist psychiatric institution; no previous use of psychiatric medications such as antidepressants, mood stabilisers or antipsychotics, or only a history of short-term, occasional use that did not constitute systematic treatment; able to complete the relevant SCL-90 independently or under the standardised guidance of research staff; possessing relatively complete records of the initial consultation and willing to undergo subsequent follow-up; and the patient has given informed consent and is voluntarily participating in this study.

Furthermore, to ensure the scientific rigour and integrity of the study, patients with psychiatric symptoms attributable to clearly defined neurological disorders, organic brain disorders, severe physical illnesses or medication-related factors are excluded; patients with clearly defined substance-induced or substance-related mental disorders; those with severe intellectual disability, cognitive impairment or impaired consciousness who were unable to complete baseline data collection and the SCL-90; those in a state of severe agitation, impulsivity, catatonia or any other condition rendering them unable to cooperate with the study assessments; those with significant gaps in their initial consultation records or an incomplete SCL-90; those who did not complete the prescribed follow-up and for whom longitudinal diagnostic outcomes could not be obtained; and those deemed by the investigator to have other conditions rendering them unsuitable for inclusion in this study.

The prevalence of each target diagnostic label and the distribution of patients without any positive target label are presented in **Table 1**.

**Table 1:** Prevalence of the target diagnostic labels in the model-development cohort.

Diagnostic label	Positive cases	Prevalence
Depression	1655	28.5%
Bipolar disorder	1260	21.7%
Anxiety disorder	1803	31.1%
Obsessive-compulsive disorder	1285	22.1%
Psychotic disorder	1086	18.7%
At least one positive target label	3243	55.9%
No positive target label	2559	44.1%

The diagnostic labels are not mutually exclusive, as a single patient may have multiple diagnosed mental disorders. Consequently, the sum of the patient counts and percentages across these labels exceeds the total number of patients or 100%.

### 2.2 Dataset structure

#### 2.2.1 Baseline DI and CHI characteristics

Baseline data were collected from all patients before their first treatment session, and all assessments were conducted according to standardised procedures by trained researchers. The baseline data comprised demographic information (DI), clinical history information (CHI) and SCL-90 assessments. The demographic and clinical-history characteristics of the patients are summarised in **Table 2** using mean ± standard deviation for continuous variables and n (%) for categorical variables.

**Table 2:** Baseline DI, CHI, and SCL-90 characteristics of first-visit psychiatric patients.

Category	Variable	Clinical relevance	Category	n (%) or mean ± SD
DI	age	reflects disease risk differences across age groups	Overall	34.6 ± 10.2
			18-27	1857 (32.0%)
			28-36	1683 (29.0%)
			37-45	870 (15.0%)
			46-55	1392 (24.0%)
	sex	evaluates differences in disease distribution between sexes	Male	2205 (38.0%)
			Female	3597 (62.0%)
	education level	evaluates the influence of education level on SCL-90 comprehension and responses	Primary school	696 (12.0%)
			Junior high school	1102 (19.0%)
			Senior high school	1335 (23.0%)
			College or above	2669 (46.0%)
	marital status	reflects social and family support conditions	Single	928 (16.0%)
			Married	2611 (45.0%)
			Divorced	1857 (32.0%)
			Widowed	406 (7.0%)
	employment status	evaluates social functioning and living status	Employed	2785 (48.0%)
			Student	1683 (29.0%)
			Unemployed	1044 (18.0%)
			Retired	290 (5.0%)
	living arrangement	reflects daily support and caregiving conditions	Living alone	3075 (53.0%)
			Living with family	2727 (47.0%)
CHI	age at onset	evaluates early- or late-onset disease characteristics	N/A	26.8 ± 7.4
	current symptom duration	reflects the duration of the current episode	N/A	8.6 ± 5.7
	family history of psychiatric disorders	evaluates genetic and familial risk factors	Yes	1741 (30.0%)
			No	3249 (56.0%)
			Unclear	812 (14.0%)
	substance use history	identifies or excludes substance-related psychiatric symptoms	Yes	1160 (20.0%)
			No	4177 (72.0%)
			Unclear	465 (8.0%)
	previous suspected psychotic symptoms	assists in identifying schizophrenia spectrum disorders	Yes	1160 (20.0%)
			No	3945 (68.0%)
			Unclear	697 (12.0%)
	previous suspected manic or hypomanic symptoms	assists in identifying bipolar disorders	Yes	2205 (38.0%)
			No	2553 (44.0%)
			Unclear	1044 (18.0%)
	previous suspected depressive episodes	assists in identifying depressive disorders	Yes	3713 (64.0%)
			No	1160 (20.0%)
			Unclear	929 (16.0%)
	previous significant anxiety or panic symptoms	assists in identifying anxiety disorders	Yes	3017 (52.0%)
			No	1973 (34.0%)
			Unclear	812 (14.0%)
	previous dissociative or conversion symptoms	assists in identifying dissociative and conversion disorders	Yes	1276 (22.0%)
			No	3597 (62.0%)
			Unclear	929 (16.0%)
	previous obsessive-compulsive symptoms	assists in identifying obsessive-compulsive disorder	Yes	1973 (34.0%)
			No	2785 (48.0%)
			Unclear	1044 (18.0%)

#### 2.2.2 SCL-90 assessment and item-level symptom characteristics

The SCL-90 is a widely used self-report psychological assessment tool for evaluating multidimensional psychiatric symptoms(20). It consists of 90 items covering multiple symptom domains, including somatization, obsessive-compulsive symptoms, interpersonal sensitivity, depression, anxiety, hostility, phobic anxiety, paranoid ideation, psychoticism, and additional symptoms (21). In this study, the SCL-90 was used to characterize patients’ symptom profiles at the item level and served as the symptom feature input for model development.

Each SCL-90 item was scored using a five-point Likert scale, where scores of 1–5 represent “none, “ “mild, “ “moderate, “ “moderately severe, “ and “severe, “ respectively (22). The total score was calculated by summing the scores of all 90 items, ranging from 90 to 450. The calculation method is shown in Equations 1–3:

(1) $$F_{j}=\frac{\sum_{i=1}^{n_{j}}X_{ij}}{n_{j}}$$

(2) $$T=\sum_{i=1}^{90}X_{i}$$

(3) $$\bar{X}=\frac{T}{90}$$

where, *F_j_* represents the factor mean for the *j*-th factor, *X_i_* represents the score for the *i*-th item, *X_ij_* represents the score for the *i*-th item within the j-th factor, nj represents the number of items included in the *j*-th factor, *T* represents the SCL-90 total score, and $\bar{X}$ represents the SCL-90 mean score.

The validated Chinese version of the SCL-90 was used, and internal consistency was assessed using Cronbach’s α. Because its 1–5 scoring system differs from the commonly used 0–4 system, the thresholds shown in the figures are specific to the scoring system used in this study.

The composition of the ten SCL-90 symptom factors and their corresponding assessment domains is summarised in **Table 3**, and the corresponding symptom severity levels are presented in **Figure 1**. **Figure 2** shows the factor-score distributions of patients with at least one target diagnosis. Because displaying all patients would result in excessive point overlap, 50 patients were randomly selected using a fixed random seed of 42 for visualisation. The 2559 patients without any target diagnosis are not displayed in **Figure 2** but were retained in all model analyses.

**Table 3:** Composition and assessment content of the SCL-90 factors.

Factor	Number of items	Main assessment domain	Typical manifestations
Somatization	12	physical discomfort and somatic manifestations of anxiety	headache, chest pain, dizziness, gastrointestinal discomfort, muscle pain
obsessive-compulsive symptoms	10	uncontrollable thoughts, impulses, or behaviors	Repeated checking, repetitive hand washing, persistent meaningless thoughts
interpersonal sensitivity	9	feelings of discomfort, inferiority, and self-consciousness in interpersonal interactions	Feeling inferior to others, being easily hurt, feeling misunderstood
depression	13	depressive mood and related cognitive and behavioral symptoms	Depressed mood, loss of interest, feelings of worthlessness, hopelessness
anxiety	10	tension, irritability, panic, and associated physical responses	Palpitations, trembling, restlessness, sudden fear
hostility	6	hostile emotions, irritability, and aggressive impulses	Easily losing temper, arguments, throwing objects, thoughts of harming others
phobic anxiety	7	excessive fear of specific places or situations	Fear of going out alone, fear of crowds, fear of transportation
paranoid ideation	6	suspicion, hostility, and persecutory experiences	Feeling monitored, distrust of others, believing others intend to take advantage
psychoticism	10	abnormal perception, thought disturbances, and social detachment experiences	Hearing voices that others cannot hear, feeling controlled by thoughts
additional symptoms	7	sleep, appetite, and other miscellaneous psychological problems	Difficulty falling asleep, early awakening, poor sleep quality, appetite changes

**Figure 1:**
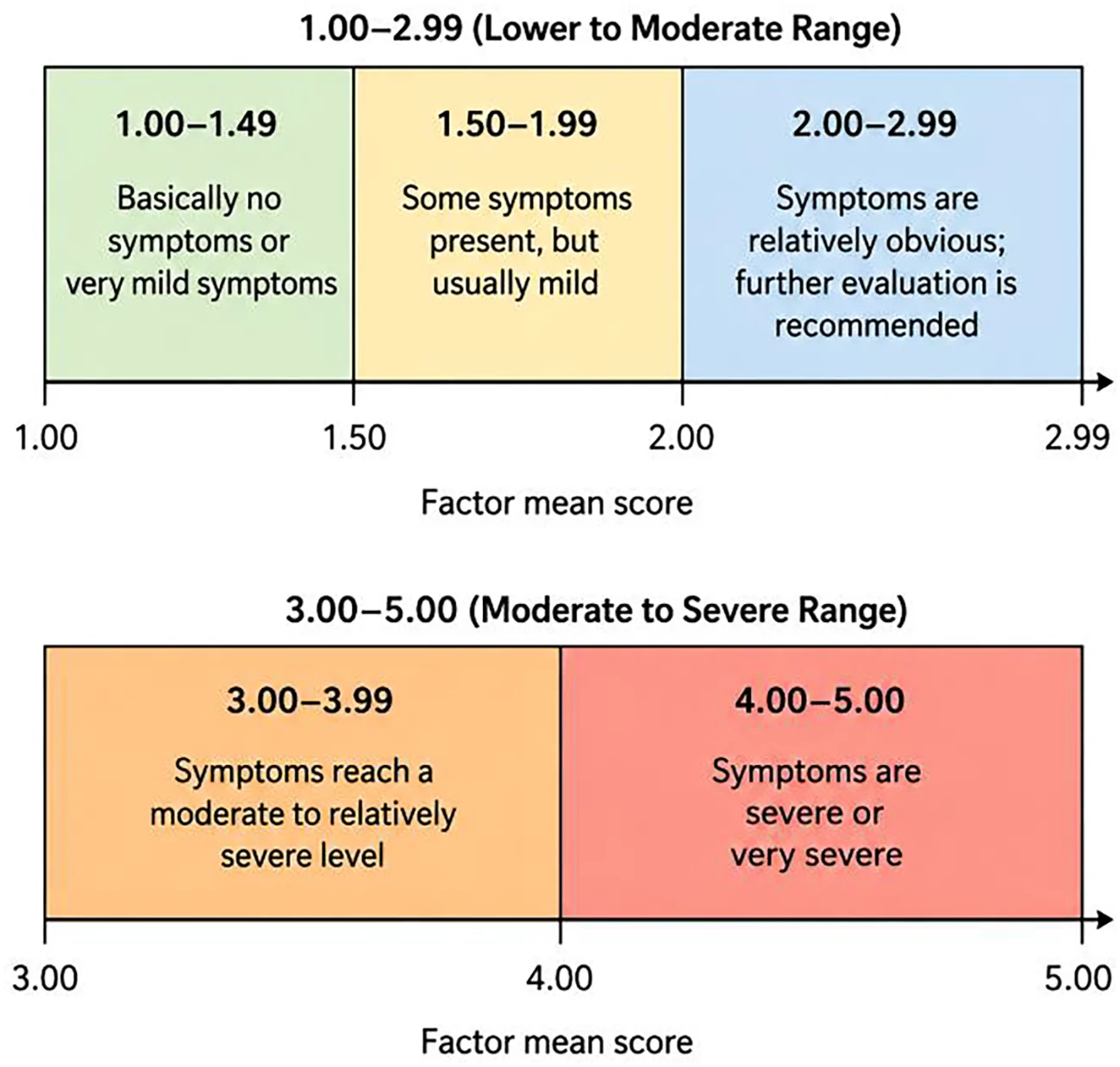
The severity levels of symptoms corresponding to each score.

**Figure 2:**
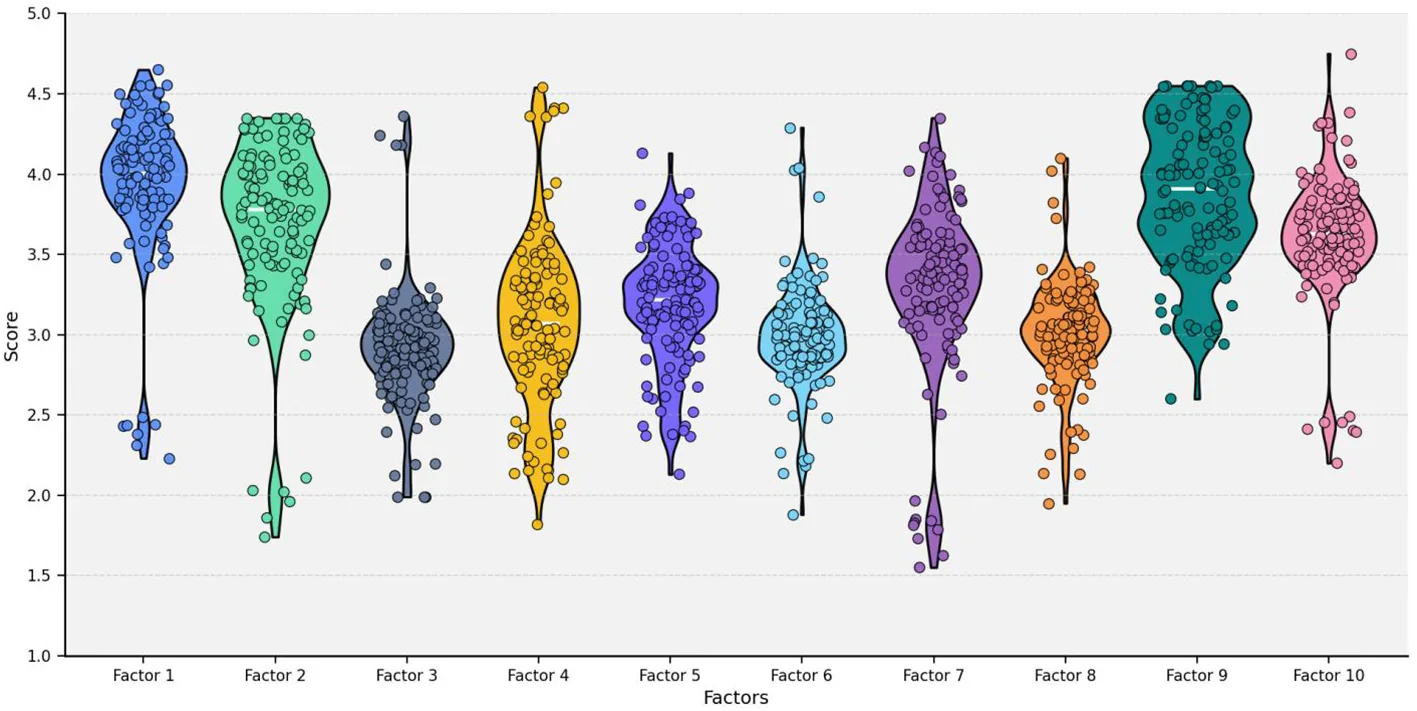
The distribution of factor mean scores among the enrolled patients.

### 2.3 Follow-up and determination of diagnostic outcomes

All enrolled patients were followed up longitudinally from their first consultation and received treatment and regular follow-up appointments as clinically indicated. Follow-up assessments included changes in affective symptoms, manic or hypomanic symptoms, psychotic symptoms, social functioning, medication adjustments, treatment response, adverse reactions, relapse and hospitalisation. Follow-up data were primarily derived from outpatient records, inpatient medical records, repeat SCL-90 scores and, where necessary, telephone follow-ups.

During the initial treatment phase, patients attended their first follow-up appointment within 3 to 7 days, at which point the treatment plan was adjusted based on changes in symptoms, treatment response and adverse reactions; a second assessment was conducted approximately 15 days later, followed by two to three further treatment assessments depending on the patient’s condition. Once the condition had stabilised, follow-ups were generally conducted every three months. At each follow-up, SCL-90 scores, clinical presentation, changes in diagnosis and treatment-related information were recorded.

The SCL-90 is re-administered at the initial consultation and at each follow-up visit to assist in reflecting the severity and trends of symptoms; it is not used as a standalone basis for diagnosis. The final diagnostic labels were independently evaluated by five psychiatrists based on longitudinal clinical data, symptom progression, treatment response and changes in social functioning. Inter-rater agreement was assessed separately for each disease label using Fleiss’ kappa. Any diagnostic disagreements were resolved through panel discussion, and the final consensus diagnosis was used as the ground-truth label.Only information collected at the initial consultation, including baseline SCL-90 item scores, demographic information and clinical history, was used as input to the model. Follow-up SCL-90 scores and other follow-up information were used exclusively to support expert determination of the final diagnostic labels and were not included as model input features during either training or validation. Therefore, no information obtained after the initial consultation was available to the model when predicting the initial-visit diagnostic outcome.

### 2.4 Model construction

Data on psychiatric disorders—such as demographic information, clinical histories or the SCL-90—exhibit typical characteristics of heterogeneous data. There are marked differences between data sources in terms of variable format, clinical significance and reliability of information, and there is significant overlap in symptoms across different disorders.

FT-Transformer is capable of converting continuous and categorical variables into feature tokens of a unified dimension, and utilises the Transformer architecture to learn non-linear relationships between different tabular variables (19). However, it typically places all variables within a single sequence, without explicitly distinguishing between different sources of clinical information, and is unable to directly handle ‘ambiguous’ medical histories, hierarchical relationships between disorders, or the need for follow-up targeted questioning.

To address these limitations, this study constructed TUCH-FT using the FT-Transformer as the backbone. The name TUCH-FT represents the four principal components added to the FT-Transformer: three-branch clinical feature encoding (TCFE), uncertainty-aware embedding (UE), cross-group multi-head attention fusion (CMAF) and hierarchical multi-label classification (HMC). The overall architecture is shown in **Figure 3**.

**Figure 3:**
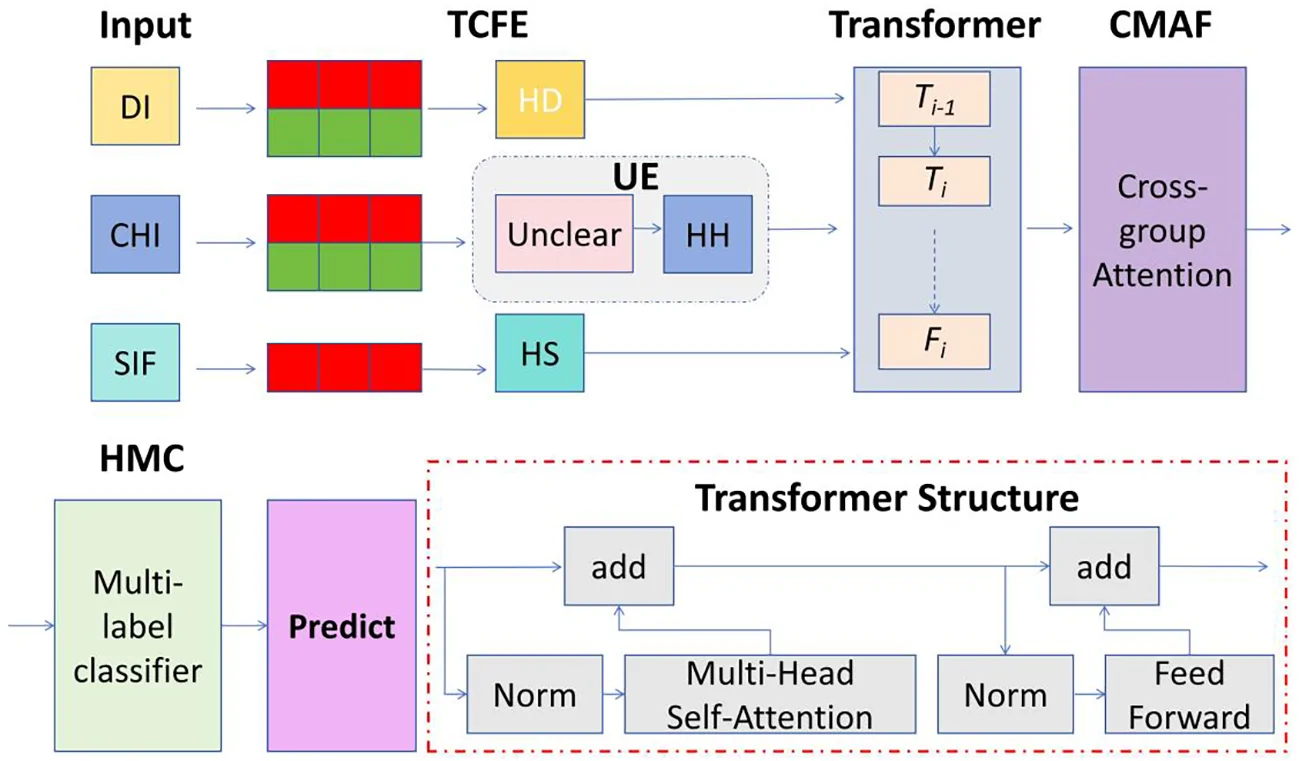
Overall structure of the TUCH-FT model.

#### 2.4.1 TCFE method

FT-Transformer processes numerical and categorical variables separately via a Feature Tokeniser. The *j* input features *x*_j_ are transformed into feature tokens *T_j_* via feature-specific mapping functions *f_j_* and bias terms *b_j_*. All feature tokens are subsequently fed into the same Transformer encoder. This method enables the unified processing of continuous and categorical variables, forming a crucial foundation for the applicability of FT-Transformer to clinical tabular data.

However, the original method did not explicitly utilise the group-level information already present amongst clinical variables. Demographic data primarily describe patients’ background risk factors, clinical history reflects the longitudinal progression of the disease and characteristics of previous episodes, whilst the SIF reflects patients’ current symptoms and their severity through individual scores for each of its 90 items. If these three types of information were placed directly within the same sequence, the model would, whilst capable of learning correlations between variables through self-attention, potentially weaken the structure within the same clinical feature group, causing a small number of clinically significant medical history variables to be diluted by a larger number of scale variables.

Therefore, building upon the feature tokenisation of the FT-Transformer, this study divides the input variables into three relatively independent branches according to their clinical origin: the demographic data branch, the CHI, and the branch of SIF. Each of these three branches performs feature tokenisation on numerical and categorical variables, respectively, and uses its own encoder to learn the relationships between variables within the group. The *j*-th feature in the FT-Transformer is defined as Equation 4:

(4) $$T_{j}=b_{j}+f_{j}\left(x_{j}\right)$$

where, x_j_ denotes the *j*-th input feature, *f_j_* denotes the mapping function corresponding to the feature type, *b_j_* is the trainable bias associated with that feature, and *T_j_* is the generated feature label. For numerical variables, *f*_j_(*x_j_*) can be expressed as an element-wise product between the numerical value and a trainable vector; for categorical variables, *f_j_*(*x_j_*) represents the vector retrieved from the corresponding category embedding table.

Once the feature segmentation was complete, the three branches generated demographic features *H_D_*, medical history features *H_H_* and SIF features HS respectively. This structure preserves the internal correlations within each group of variables prior to feature fusion, preventing the large number of SIF features from causing excessive interference with medical history and demographic variables. It also better aligns with the process followed by clinicians, who assess a patient’s background, disease course and current symptoms separately before making a comprehensive judgement. The three-branch feature encoding structure is shown in **Figure 4**.

**Figure 4:**
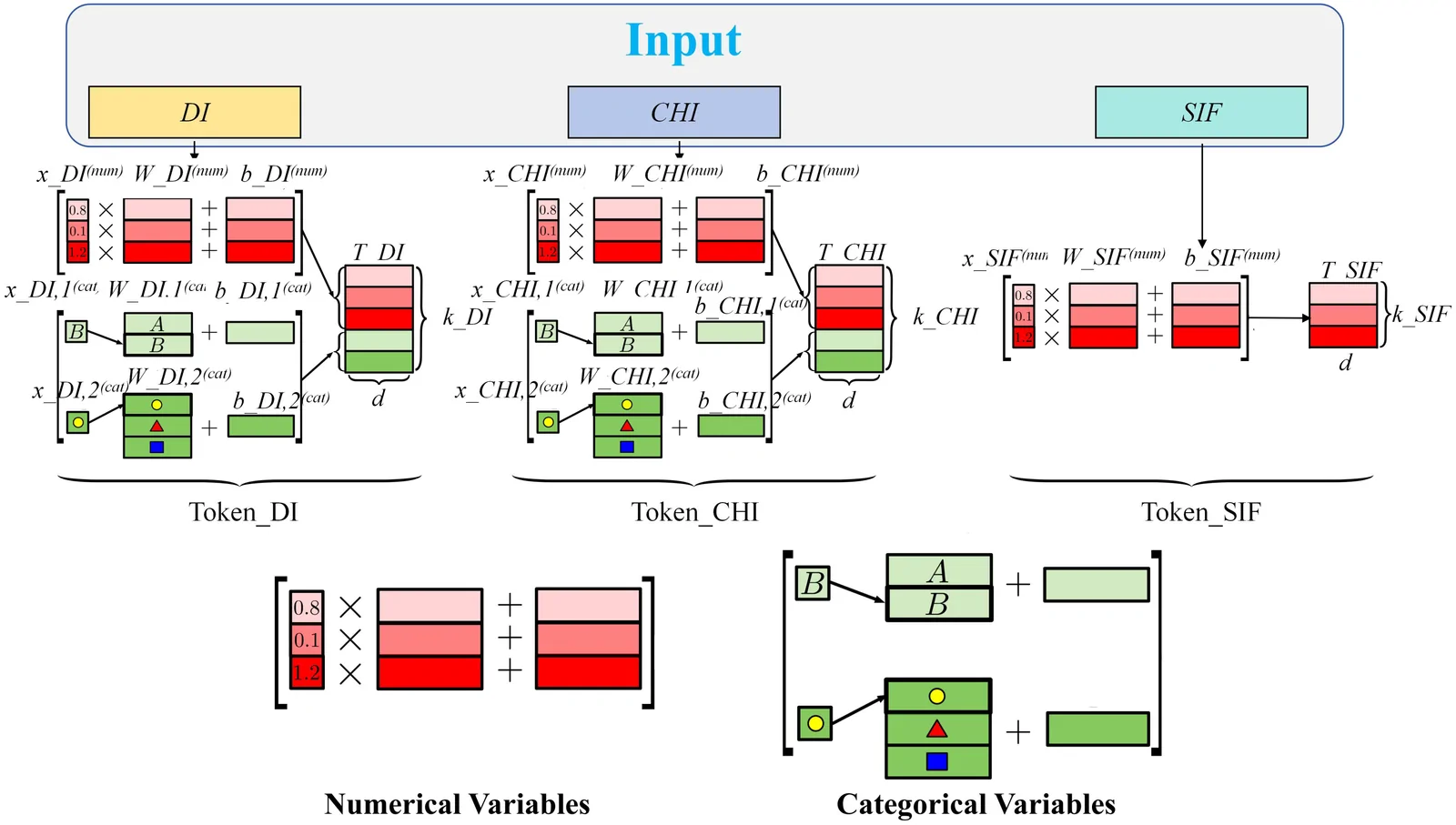
Schematic diagram of the TCFE method.

#### 2.4.2 UE module

At the initial psychiatric consultation, patients and their families may be unable to accurately identify past symptoms; consequently, the clinical history often contains entries marked as ‘unclear’. ‘Unclear’ does not equate to ‘none’, but may reflect recall bias on the part of the patient or a lack of understanding of the illness. Clinical practice has shown that such uncertain information may itself hold certain clinical significance, as it is related to insight, cognitive function, symptom duration and disease severity, and can serve as supplementary information for differential diagnosis and severity assessment. If treated simply as a standard category, the model would struggle to distinguish between “symptoms do not exist” and “information has not yet been confirmed”, and would also overlook the potential diagnostic value inherent in the uncertainty of the information itself.

To retain this type of information, this study decomposes medical history variables with an ‘unclear’ option into two channels—medical history status sj and information clarity mj—and performs embedding on each separately, the calculation method is shown in Equation 5:

(5) $$T_{j}^{H}=E_{j}^{s}\left(s_{j}\right)+E_{j}^{m}\left(m_{j}\right)$$

where, $E_{j}^{s}$ denotes the medical history state embedding, $E_{j}^{m}$ denotes the degree of certainty embedding, and $T_{j}^{H}$ denotes the joint medical history feature label.

A single indicator can signal that a history item is unclear, whereas the learned gate allows the contribution of clarity information to vary with the joint representation of history status and clarity.Building on this representation, a lightweight gating unit was introduced to adaptively regulate the contribution of information clarity. The gating vector was calculated as. The calculation method is shown in Equations 6, 7:

(6) $$g_{j}=\sigma \left(W_{g}\left[E_{j}^{s}\left(s_{j}\right)∥E_{j}^{m}\left(m_{j}\right)\right]+b_{g}\right)$$

and the final uncertainty-aware representation was obtained as:

(7) $${\tilde{T}}_{j}^{H}=LayerNorm\left[E_{j}^{s}\left(s_{j}\right)+g_{j}⊙E_{j}^{m}\left(m_{j}\right)\right]$$

where *g_j_* denotes the learnable gating vector; *W_g_* and *b_g_* are the trainable weight matrix and bias vector, respectively; σ(·) denotes the sigmoid activation function; ‖ denotes vector concatenation; and ⊙ denotes element-wise multiplication. The gating vector has the same dimensionality as the embeddings, allowing the contribution of information clarity to be adjusted independently across feature dimensions. The resulting representation ${\tilde{T}}_{j}^{H}$ is subsequently used as the input feature of the *j*-th medical history variable.

For medical histories that have been definitively confirmed, relatively stable weights are obtained, whilst for those that are unclear, their uncertainty is retained and their influence is dynamically adjusted according to the joint medical history status and information clarity representations. This structure not only avoids information bias caused by simple interpolation but also provides a basis for subsequent targeted questioning.

The dual-channel perception embedding structure for cases where the clinical history is unclear is shown in **Figure 5**.

**Figure 5:**
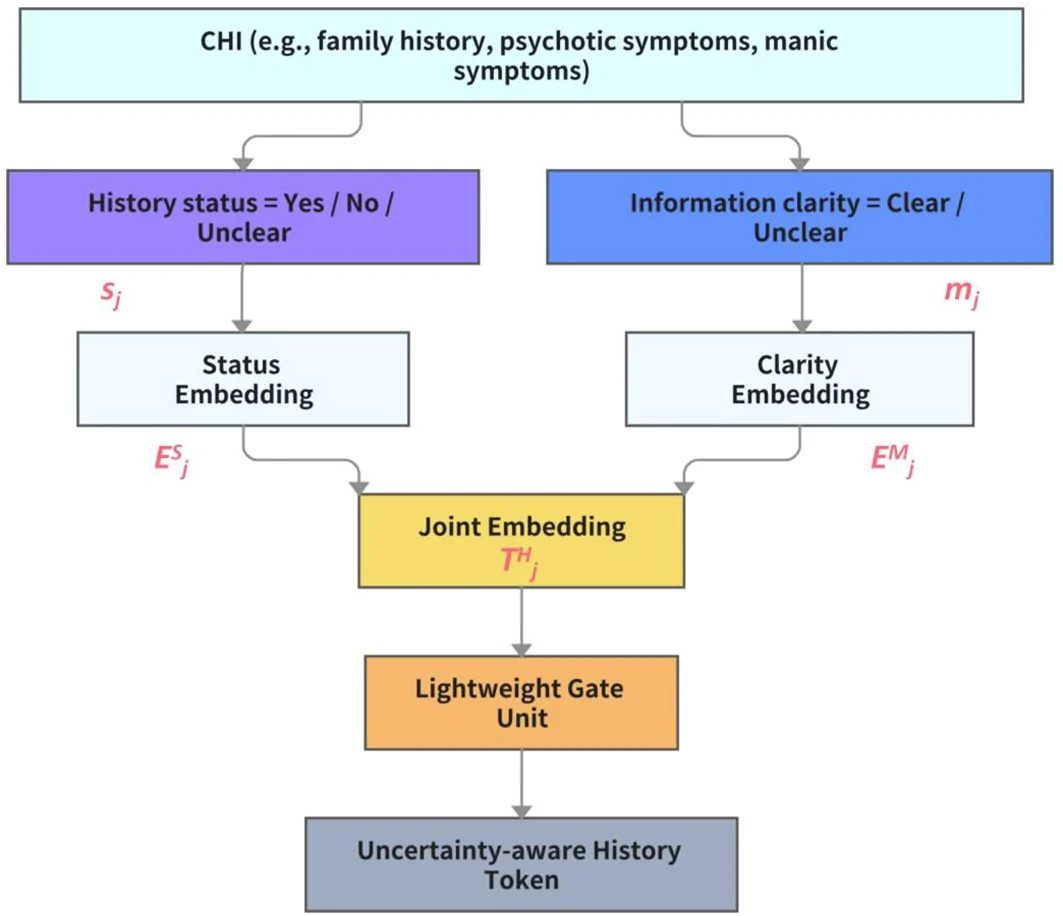
Architecture of the uncertainty-aware embedding (UE) module.

#### 2.4.3 CMAF mechanism

Following the completion of TCFE encoding, it is necessary to further investigate the interactive relationships between DI, CHI and SIF. In a standard self-attention mechanism, *Q*, *K* and *V* all originate from the same set of features, making it difficult to explicitly represent the directed retrieval relationship of one set of clinical information with respect to other sets. Therefore, this study introduces a cross-group multi-head attention mechanism, whereby one feature set acts as a query to extract relevant information from other feature sets (23). Scaled dot-product attention is defined by Equation 8, whilst multi-head attention is represented by Equations 9, 10:

(8) $$\text{Attention}\left(Q,K,V\right)=\text{softmax}\left(\frac{QK^{T}}{\sqrt{d_{k}}}\right)V$$

(9) $$\text{MultiHead}\left(Q,K,V\right)=\text{Concat}\left({\mathrm{head}}_{1},\ldots ,{\mathrm{head}}_{\text{h}}\right)W^{O}$$

(10) $$head_{i}=\text{Attention}\left(QW_{i}^{Q},KW_{i}^{K},VW_{i}^{V}\right)$$

where, $Q\in R^{n_{q}\times d_{\text{model}}}$, $K\in R^{n_{k}\times d_{\text{model}}}$ and $V\in R^{n_{k}\times d_{\text{model}}}$, denote the query, key and value matrices, respectively; *n_q_* denotes the number of query tokens; *n_k_* denotes the number of key/value tokens; *d_model_* denotes the feature dimension; and *h* denotes the number of attention heads. For each attention head, *d_k_*=*d_v_*=*d_model_*/*h*, The projection matrices satisfy, $W_{i}^{Q},\;W_{i}^{K}\in R^{d_{\text{model}}\times d_{k}}$, and $W_{i}^{V}\in R^{d_{\text{model}}\times d_{v}}$, Therefore, each head*_i_* has dimensions *n_q_* × *d_v_*, and the concatenated multi-head representation has dimensions *n_q_* × *h_dv_*. The output projection matrix $W^{O}\in R^{hd_{v}\times d_{\text{model}}}$, maps the concatenated representation back to *n_q_* × *d_model_*. Here, *W°* is shared after the concatenation of all attention heads and therefore does not contain the head-specific subscript *i*. Concat(·) denotes feature concatenation.

In this paper, three cross-group attention directions are defined. When *H_D_* serves as the query, the model extracts information related to DI from *H_H_* and *H_S_*; when *H_H_* serves as the query, the model extracts information related to past medical history from DI and SIF; when *H_S_* serves as the query, the model extracts information from DI and CHI that can explain the current symptom pattern.

The outputs of the three directed cross-group attention branches were mean-pooled and concatenated, and then projected into a unified fused representation, the calculation method is shown in Equation 11:

(11) $$H_{F}=LayerNorm\left(W_{F}\left[MeanPool\left(H_{D}^{\prime }\right)\left\|MeanPool\left(H_{H}^{\prime }\right)\right\|MeanPool\left(H_{S}^{\prime }\right)\right]+b_{F}\right)$$

where $H_{D}^{\prime }$, $H_{H}^{\prime }$ and $H_{S}^{\prime }$ denote the outputs of the DI-, CHI- and SIF-directed cross-group attention branches, respectively; MeanPool(·) denotes mean pooling over the token dimension; ‖ denotes feature concatenation; and *W_F_* and *b_F_* are the trainable projection matrix and bias, respectively. The resulting fused representation *H_F_* was subsequently passed to the hierarchical classification heads.

**Figure 6** illustrates the aforementioned cross-group feature fusion structure.

**Figure 6:**
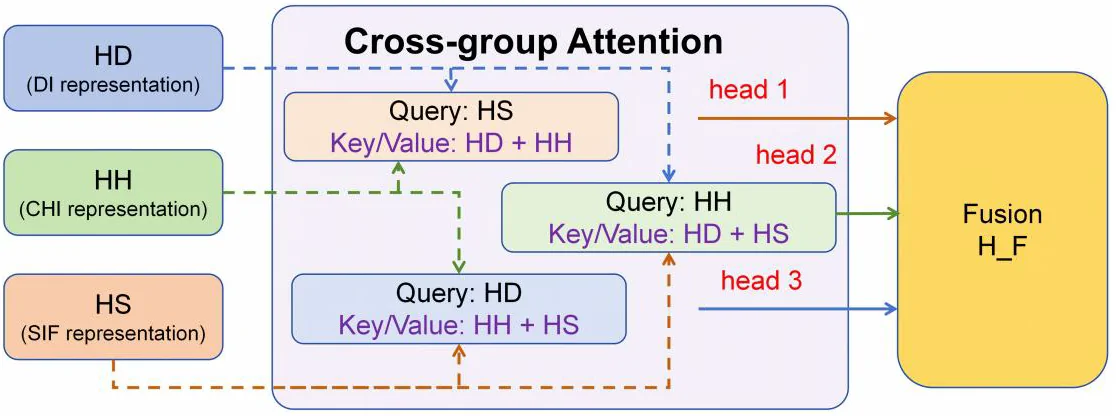
Principles of the CMAF mechanism and a cross-group fusion structure of three sets of clinical features.

#### 2.4.4 HMC structure

Patients with mental disorders may have one or more comorbid conditions at the time of clinical diagnosis, and there may be comorbidity or associations between different conditions. For example, patients with depressive disorders may also present with anxiety disorders, obsessive-compulsive symptoms or other mental disorders. Therefore, this study does not restrict each patient to a single disease category; instead, the diagnostic task is formulated as a hierarchical multi-label classification, enabling the model to simultaneously output predicted weights for multiple conditions.

The broad disease categories were defined as affective disorders, anxiety-related disorders and psychotic disorders. Affective disorders comprised depressive disorder and bipolar disorder; anxiety-related disorders comprised anxiety disorder and obsessive-compulsive disorder; and psychotic disorders comprised psychotic disorder. Accordingly, each specific disease label was linked to its corresponding parent category for the calculation of the hierarchical consistency loss.

In this study, the model incorporates separate prediction heads for broad disease categories and specific diseases, and the relationship between higher-level disease categories and specific diseases is constrained by a hierarchical consistency loss. Unlike single-label classification, where the sum of probabilities across all categories must equal 1, this study adopts a multi-label output approach, with each disease having a relatively independent prediction probability. The prediction weights for the cth disease are expressed as Equation 12, and the total model loss is defined as Equation 13:

(12) $$p_{c}=\sigma \left(z_{c}\right)$$

(13) $$L=L_{d}+\lambda_{g}L_{g}+\lambda_{c}L_{c}$$

where, *z_c_* represents the model output corresponding to the *c*-th disease; σ(·) is the sigmoid activation function; and *p_c_* ∈ [0, 1] denotes the prediction weight indicating whether the patient has that disease. *L_d_* is the multi-label classification loss for a specific disease, *L_g_* is the multi-label classification loss for a broad disease category, *L_c_* is the hierarchical consistency loss, *λ_g_* and *λ_c_* are the corresponding weight coefficients. They were selected by grid search on the validation set from {0.1, 0.3, 0.5, 1.0}, using Macro-F1 as the selection criterion, and were finally set to 0.5 and 0.1, respectively. Both broad disease category prediction and specific disease prediction utilise binary cross-entropy loss, enabling each disease to be independently classified as positive or negative.

As the probabilities of different diseases are mutually independent, a single patient may simultaneously receive multiple high disease prediction weights. The hierarchical consistency loss is used to constrain the relationship between a specific disease and its corresponding broad disease category, the calculation method is shown in Equation 14:

(14) $$L_{c}=\frac{1}{C}\sum_{c=1}^{C}\max\left(0,p_{c}^{d}-p_{\pi \left(c\right)}^{g}\right)$$

where *C* is the number of specific diseases, $p_{c}^{d}$ is the predicted probability of the *c*-th specific disease, $p_{\pi \left(c\right)}^{g}$ is the predicted probability of its corresponding parent disease category, and π(*c*) denotes the mapping from a specific disease to its parent category. Thus, a penalty is imposed when the predicted probability of a specific disease exceeds that of its parent category.

The model’s final output is a disease profile comprising multiple diseases and their respective prediction weights. Diseases with prediction weights exceeding a set threshold may be listed as candidate positive diagnoses; diseases that have not yet reached the positive threshold but have relatively high prediction weights may be listed as candidate conditions requiring further differentiation or follow-up confirmation. Consequently, the model is able to simultaneously reflect the primary disease, possible co-existing conditions and diseases requiring further investigation, rather than forcing complex psychiatric symptoms into a single category. The hierarchical multi-label classification structure is shown in **Figure 7**.

**Figure 7:**
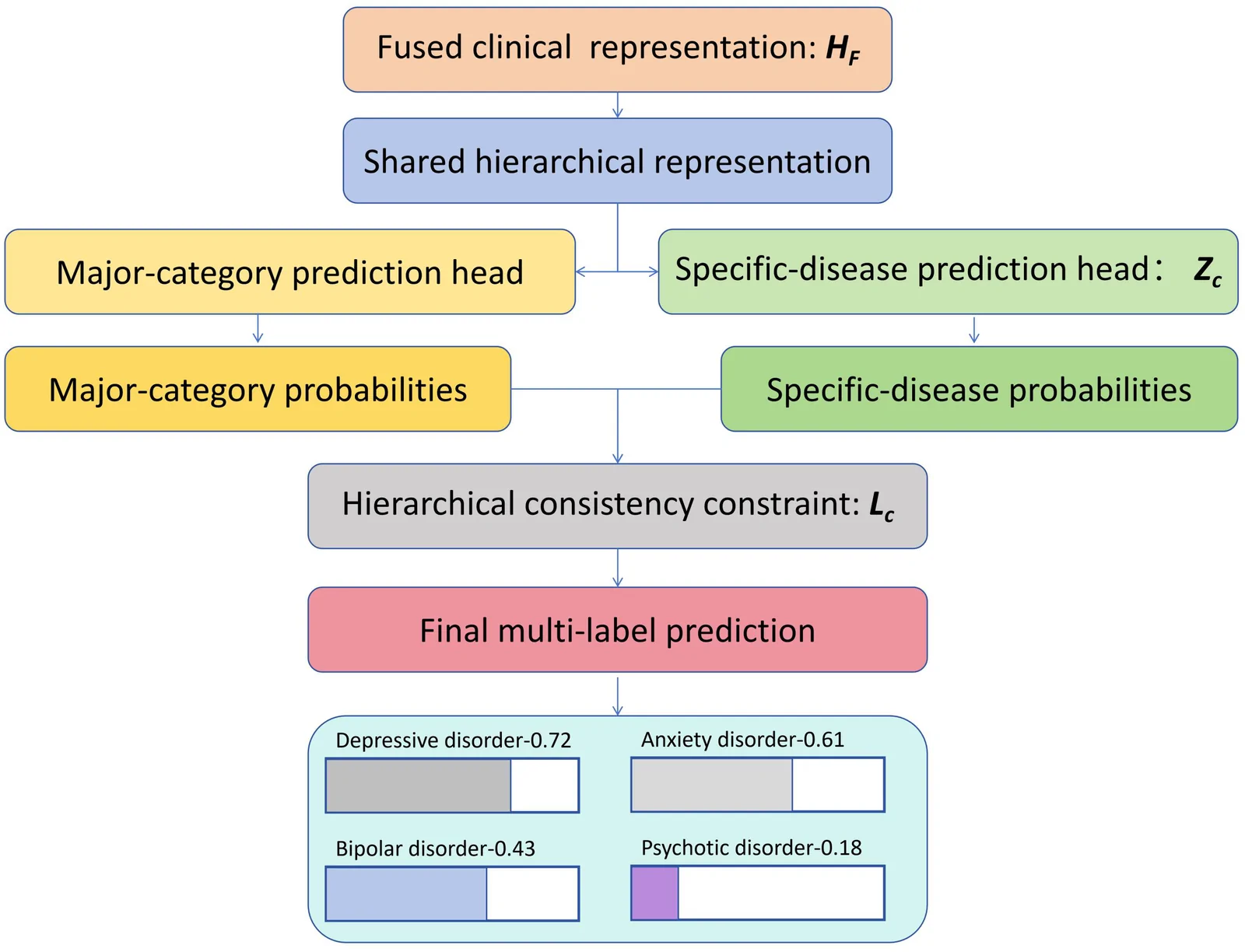
Schematic diagram of the HMC structure.

### 2.5 Data partitioning and model development

The 5802 patients in the model-development cohort were divided at the patient level into training and validation sets at a ratio of 7:3 (4061/1741) using multilabel-stratified splitting, with no patient overlap. The training set was used for parameter estimation, whereas the validation set was used for model selection, hyperparameter tuning and decision-threshold determination. A temporally separated cohort of 1272 patients was subsequently recruited from the same hospital between January 2024 and January 2025. This cohort had no patient overlap with the development cohort and was not used for model training, model selection, hyperparameter tuning or threshold determination. It was used to assess the discrimination and calibration of the fixed model. Because both cohorts originated from the same institution, this evaluation represents temporal same-centre validation rather than external validation.

The model was trained using AdamW with an initial learning rate of 1×10^−4^, a batch size of 64, a weight decay of 1×10^−5^, and a maximum of 300 epochs. Early stopping with a patience of 10 epochs was based on validation loss. The embedding dimension, Transformer layers, attention heads and dropout rate were 128, 3, 8 and 0.1, respectively. Class-weighted binary cross-entropy was used, with weights calculated from the training set. Label-specific thresholds were selected from 0.05 to 0.95 in increments of 0.01 by maximising validation-set F1 and were fixed for subsequent evaluations. Experiments were implemented using Python 3.10.13, PyTorch 2.1.2 and scikit-learn 1.3.2 on an NVIDIA RTX 3090 GPU with CUDA 12.1. TUCH-FT contained approximately 3.1 million trainable parameters. The mean training time was approximately 11.4 seconds per epoch and 31.6 minutes per complete run, while the mean inference time was 2.1 ms per patient using a batch size of 1.

All models were independently evaluated using five random seeds (42, 3407, 2024, 225 and 346), with results reported as the mean ± standard deviation. The calculation method is shown in Equation 15:

(15) $$\bar{M}\pm \text{SD}=\frac{1}{S}\sum_{s=1}^{S}M_{s}\pm \sqrt{\frac{1}{S-1}\sum_{s=1}^{S}{\left(M_{s}-\bar{M}\right)}^{2}}$$

In addition, 95% confidence intervals were calculated from 1000 bootstrap resamples of the test set using the percentile method. The calculation method is shown in Equation 16:

(16) $${\text{CI}}_{95\%}=\left[Q_{0.025}\left(M^{\left(1\right)},\ldots ,M^{\left(B\right)}\right),Q_{0.975}\left(M^{\left(1\right)},\ldots ,M^{\left(B\right)}\right)\right],B=1000$$

where *M_s_* denotes the performance metric obtained using the *s*-th random seed, and S = 5; *M*^(^*^b^*^)^ denotes the performance metric obtained from the *b*-th bootstrap sample; and *Q*_0.025_ and *Q*_0.975_ denote the 2.5th and 97.5th percentiles of the bootstrap distribution, respectively. Paired bootstrap comparisons against the FT-Transformer and between adjacent ablation configurations used identical resampled patients; differences were considered statistically supported when their 95% confidence intervals excluded zero.

Reporting followed the TRIPOD+AI guideline (24), with the completed checklist provided as **Supplementary File S2**.

## 3 Results

### 3.1 Experimental design and evaluation criteria

This study uses the scores from the DI, CHI and the SIF, collected at the patient’s first consultation, as model inputs, and encodes the disease diagnoses—as comprehensively determined by psychiatric specialists following longitudinal follow-up—into multi-hot label vectors. For each patient, a value of 1 is assigned to the position corresponding to a confirmed diagnosis, and a value of 0 to the position corresponding to an unconfirmed diagnosis; where a patient has multiple concurrent conditions, the label vector may contain multiple positive class labels.

Upon completion of model training, the model outputs the predicted probabilities of a patient belonging to each disease category. For each candidate disease, the model generates a probability value between 0 and 1 via an independent Sigmoid output node. The predicted probability for each disease was converted into a positive or negative label using the corresponding label-specific threshold selected exclusively on the validation set. Once determined, these thresholds were fixed and applied without further adjustment to the internal test set and temporal same-centre validation cohort. Consequently, the model can output both a probability ranking for each disease and a final determination as to whether the patient has the corresponding disease. When the predicted probabilities for multiple diseases simultaneously exceed their respective thresholds, the model may output multiple disease labels.

Model performance is evaluated using Macro-F1, Micro-F1, Macro-AP, Hamming Loss and Label Ranking Average Precision (LRAP). The calculation method is shown in Equations 17–21:

(17) $$\mathrm{Macro‐F}1=\frac{1}{K}\sum_{k=1}^{K}\frac{2{TP}_{k}}{2{TP}_{k}+{FP}_{k}+{FN}_{k}}$$

(18) $$\mathrm{Micro‐F}1=\frac{2\sum_{k=1}^{K}{TP}_{k}}{2\sum_{k=1}^{K}{TP}_{k}+\sum_{k=1}^{K}{FP}_{k}+\sum_{k=1}^{K}{FN}_{k}}$$

(19) $$\mathrm{Macro‐AP}=\frac{1}{K}\sum_{k=1}^{K}AP_{k}$$

(20) $$\mathrm{HammingLoss}=\frac{1}{NK}\sum_{i=1}^{N}\sum_{k=1}^{K}I\left(y_{ik}\neq {\hat{y}}_{ik}\right)$$

(21) $$LRAP=\frac{1}{N}\sum_{i=1}^{N}\frac{1}{\left|Y_{i}\right|}\sum_{k\in Y_{i}}\frac{\left|\left\{j\in Y_{i}:p_{ij}\geq p_{ik}\right\}\right|}{\left|\left\{j:p_{ij}\geq p_{ik}|\right\}\right|}$$

where, *N* represents the number of patients, *K* represents the number of disease categories, *TP_k_*, *FP_k_* and *FN_k_* represent the number of true positives, false positives and false negatives for disease category *k* respectively; *AP_k_* represents the average precision for disease category *k*, and *Y_i_* represents the set of true diseases for patient *i*.

### 3.2 Comparative tests of different models

To evaluate the performance of TUCH-FT in multi-label classification tasks for mental disorders, this study selected six models—Logistic Regression (LR), Random Forest (RF), Extreme Gradient Boosting (XGBoost), Multilayer Perceptron (MLP), FT-Transformer and TUCH-FT—for comparison. LR, RF and XGBoost employed the Binary Relevance strategy to predict each disease label respectively, whilst MLP, FT-Transformer and TUCH-FT utilised a Sigmoid multi-label output layer. The experimental results are shown in **Table 4**.

**Table 4:** A comparison of the performance of different models in multi-label classification tasks for mental disorders.

Model	Model type	Macro-F1↑	Micro-F1↑	Macro-AP↑	Hamming Loss↓	LRAP↑
LR	linear classification models	0.540	0.612	0.575	0.236	0.642
RF	ensemble tree models	0.578	0.648	0.612	0.214	0.671
XGBoost	gradient-boosted tree models	0.612	0.681	0.645	0.193	0.703
MLP	basic deep learning models	0.668	0.724	0.701	0.162	0.748
FT-Transformer	table models	0.730	0.775	0.760	0.128	0.813
TUCH-FT	the improved model proposed in this paper	0.803	0.842	0.838	0.095	0.876

“↑” indicates that higher values correspond to better performance_1_; “↓” indicates that lower values correspond to better performance_2_.

The results show that, compared with deep learning models, machine learning has limited expressive power in the multi-label mental disorder prediction task. This is because such methods rely on manually engineered features or shallow non-linear classification capabilities, making it difficult to effectively model complex higher-order interactions.

FT-Transformer achieved superior performance among all compared models, owing to its ability to model tabular data in a structured manner: by unifying the embedding of different types of variables through a feature tokenisation mechanism, and utilising self-attention mechanisms to capture global dependencies between variables, it is able to model the interactions between clinical variables more effectively.

Building on this, the proposed TUCH-FT model achieved a Macro-F1 of 0.803, Micro-F1 of 0.842, Macro-AP of 0.838, Hamming Loss of 0.095, and LRAP of 0.876. Compared with the original FT-Transformer, Macro-F1, Micro-F1, Macro-AP, and LRAP increased by 10.0%, 8.6%, 10.3%, and 7.7%, respectively, while Hamming Loss decreased by 25.8%, demonstrating the effectiveness of the proposed improvements.

### 3.3 Label-specific classification performance

To further evaluate the discrimination performance of TUCH-FT across diagnostic labels with different prevalence rates, label-specific precision, recall, F1 score, area under the receiver operating characteristic curve (AUC) and average precision (AP) were calculated in the temporal same-centre validation cohort. Macro-F1 and Macro-AP were also calculated to summarise performance across the five diagnostic labels. Exact-match accuracy was calculated to determine whether the complete multi-label prediction matched the ground-truth label combination for each patient. The results are presented in **Table 5**.

**Table 5:** Label-specific discrimination performance of TUCH-FT in the temporal same-centre validation cohort.

Diagnostic label	Positive (%)	Precision	Recall	F1 score	AUC	AP
Depression disorder	649 (51.0%)	0.838	0.812	0.825	0.902	0.854
Bipolar disorder	494 (38.8%)	0.792	0.758	0.775	0.887	0.817
Anxiety disorder	707 (55.6%)	0.844	0.821	0.832	0.915	0.872
Obsessive-compulsive disorder	504 (39.6%)	0.806	0.772	0.789	0.891	0.824
Psychotic disorder	426 (33.5%)	0.817	0.774	0.795	0.906	0.823
Macro-average	N/A	0.819	0.787	0.803	0.900	0.838

Exact-match accuracy was 0.608 (773/1272), indicating that all five predicted labels matched the reference diagnoses for 773 patients.

The prevalence of all five diagnostic labels was higher in the temporal validation cohort than in the development cohort (**Table 5** vs. **Table 1**). The reasons for this case-mix difference cannot be established from the available data. Because the label-specific thresholds were selected in the development cohort and applied unchanged to the temporal cohort, the discrimination and calibration results should be interpreted in the context of this prevalence shift.

In the temporal same-centre validation cohort, TUCH-FT achieved a Macro-F1 of 0.803 and a Macro-AP of 0.838. Across the five diagnostic labels, F1 scores ranged from 0.775 to 0.832, AUC values ranged from 0.887 to 0.915, and AP values ranged from 0.817 to 0.872. The exact-match accuracy was 0.608 (773/1272), indicating that the complete predicted label combination matched the ground-truth combination for 60.8% of patients.

Anxiety disorder and depressive disorder showed relatively strong discrimination performance, whereas bipolar disorder had the lowest recall and F1 score, indicating greater difficulty in identifying this condition. Overall, TUCH-FT maintained relatively balanced discrimination performance across diagnostic labels with different prevalence rates, although further external validation in other institutions remains necessary.

### 3.4 Experiments on different combinations of input features

To analyse the contribution of different types of clinical information to the multi-label classification results for mental disorders, this study established various combinations of input features and conducted ablation analyses; the results are shown in **Table 6**. In the table, SIF features are denoted by SIF.

**Table 6:** Results of experiments with different combinations of input features.

Features			Macro-F1↑	Micro-F1↑	Macro-AP↑	HammingLoss↓	LRAP↑
DI	CHI	SIF					
✓			0.126	0.193	0.154	0.342	0.287
	✓		0.438	0.482	0.451	0.246	0.536
		✓	0.451	0.497	0.466	0.239	0.548
✓	✓		0.701	0.742	0.719	0.146	0.781
✓		✓	0.728	0.768	0.747	0.134	0.806
	✓	✓	0.756	0.791	0.778	0.121	0.829
✓	✓	✓	0.803	0.842	0.838	0.095	0.876

The results indicate that whilst model performance improved when only CHI or SIF was input, the Macro-F1 scores were only 0.438 and 0.451 respectively, and overall performance remained at the threshold for auxiliary identification. CHI reflects a patient’s history of episodes and disease risk, whilst SIF provides information on current symptoms; however, neither source of information alone is sufficient to adequately distinguish between mental disorders with similar symptomatic presentations.

Age, gender and socio-demographic information primarily reflect background risk factors for disease onset; lacking direct evidence of symptoms and medical history, they cannot serve as independent diagnostic information. Consequently, when DI is inputted alone, it is unable to complete disease identification. However, when DI was combined with CHI, Macro-F1 improved from 0.438 to 0.701; when DI was combined with SIF, Macro-F1 improved from 0.451 to 0.728. This indicates that although DI does not possess effective disease assessment capabilities in its own right, it can provide CHI and SIF with a context for reinterpreting medical history and symptom characteristics within the specific patient’s background, thereby significantly enhancing the combined performance.

When CHI and SIF were combined as inputs, Macro-F1 and Micro-F1 reached 0.756 and 0.791 respectively, indicating a clear complementary relationship between the patient’s past clinical history and current symptom presentation. When DI, CHI and SIF were input together, the model’s performance underwent a further qualitative improvement. This suggests that only by simultaneously integrating the patient’s baseline background, past clinical history and current fine-grained symptoms can the model form a relatively comprehensive representation of clinical features and achieve reliable multi-label classification of mental disorders.

### 3.5 Comorbidity pattern analysis of TUCH-FT predictions

Because psychiatric disorders frequently exhibit overlapping symptoms and comorbid relationships, this study further analysed whether TUCH-FT could capture complex multi-label diagnostic patterns. The distribution of clinical disorder combinations and model predictions are shown in **Figure 8**. Disorders are abbreviated as D (depressive disorder), B (bipolar disorder), A (anxiety disorder), O (obsessive-compulsive disorder), and P (psychotic disorder). Of the 1272 patients, 130 with no predicted positive label were excluded from the positive combinations shown in **Figure 8**.

**Figure 8:**
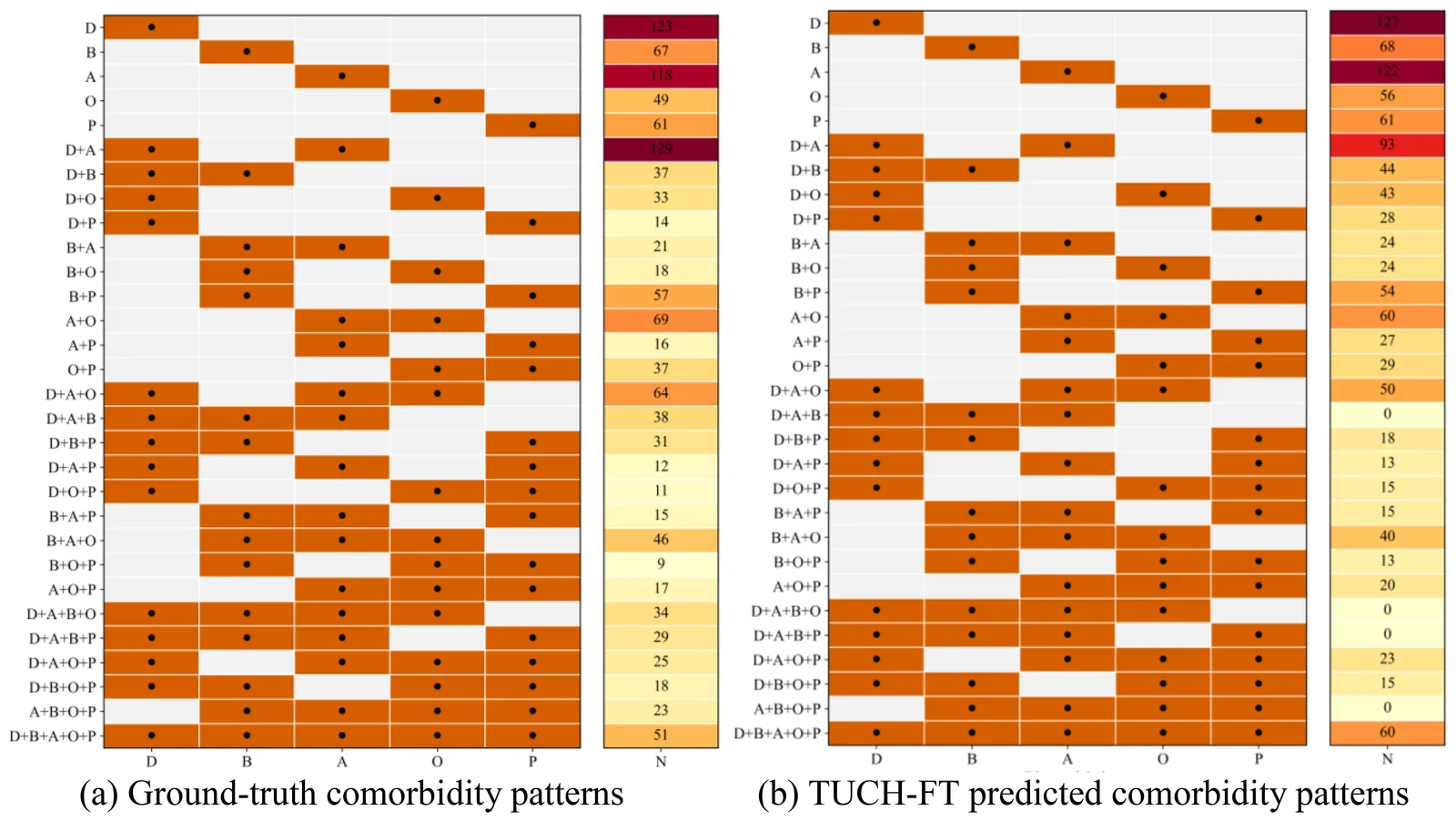
Comorbidity pattern analysis of TUCH-FT predictions.

The results showed that single disorders and two-disorder combinations represented the majority of diagnostic patterns, whereas higher-order comorbid combinations gradually decreased with increasing numbers of concurrent disorders.

TUCH-FT broadly reproduced the predominance of single-disorder and two-disorder patterns; however, discrepancies were observed for several higher-order comorbidity combinations. In particular, D+A+B was present in 38 ground-truth cases but was not predicted as an exact combination by the model. This finding indicates that TUCH-FT can capture common comorbidity patterns but remains less reliable for some complex and relatively infrequent diagnostic combinations.

To further evaluate the consistency between clinical diagnoses and model predictions at the combination level, a combination-level confusion matrix was constructed as shown in **Figure 9**. Values represent patient counts and were not row-normalised.

**Figure 9:**
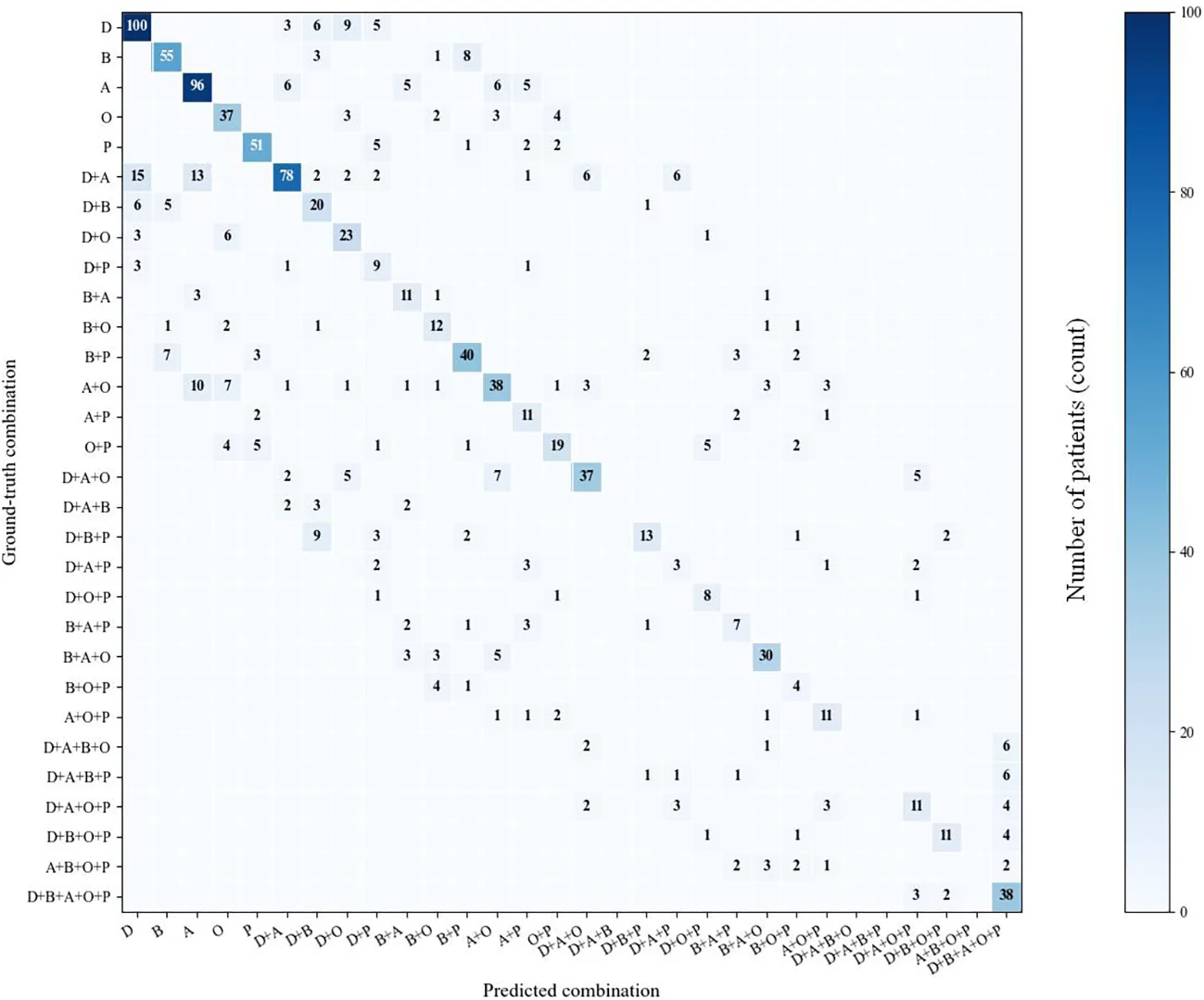
Combination-level confusion matrix between clinical diagnoses and model predictions.

Most diagnostic combinations were located along the diagonal, indicating high agreement between the predicted and clinically determined disorder patterns. Prediction errors mainly occurred among clinically related combinations, particularly between single disorders and their comorbid patterns. Among these errors, D+A was most frequently misclassified as standalone D or A, with 15 and 13 cases, respectively. This may be explained by the substantial overlap between depressive and anxiety disorders in first-visit presentations, including sleep disturbance, fatigue, impaired concentration, psychomotor changes and somatic complaints. In some patients, either depressive or anxiety symptoms may dominate the initial clinical presentation, while symptoms of the co-occurring disorder may be less prominent or remain below the model’s decision threshold. Consequently, the model may identify the dominant disorder but fail to retain the secondary label. This pattern reflects the clinical difficulty of distinguishing depression–anxiety comorbidity during initial assessment rather than random classification error.

Overall, TUCH-FT captured the major comorbidity patterns but showed reduced performance for some higher-order diagnostic combinations, indicating that its predictions of complex comorbidity structures should be interpreted cautiously.

### 3.6 Model interpretability analysis using SHAP

To further interpret the clinical information learned by TUCH-FT, SHAP (SHapley Additive exPlanations), a feature-attribution framework for interpreting model predictions, was applied from both global and disease-specific perspectives (**Figures 10**, **11**) (25).

**Figure 10:**
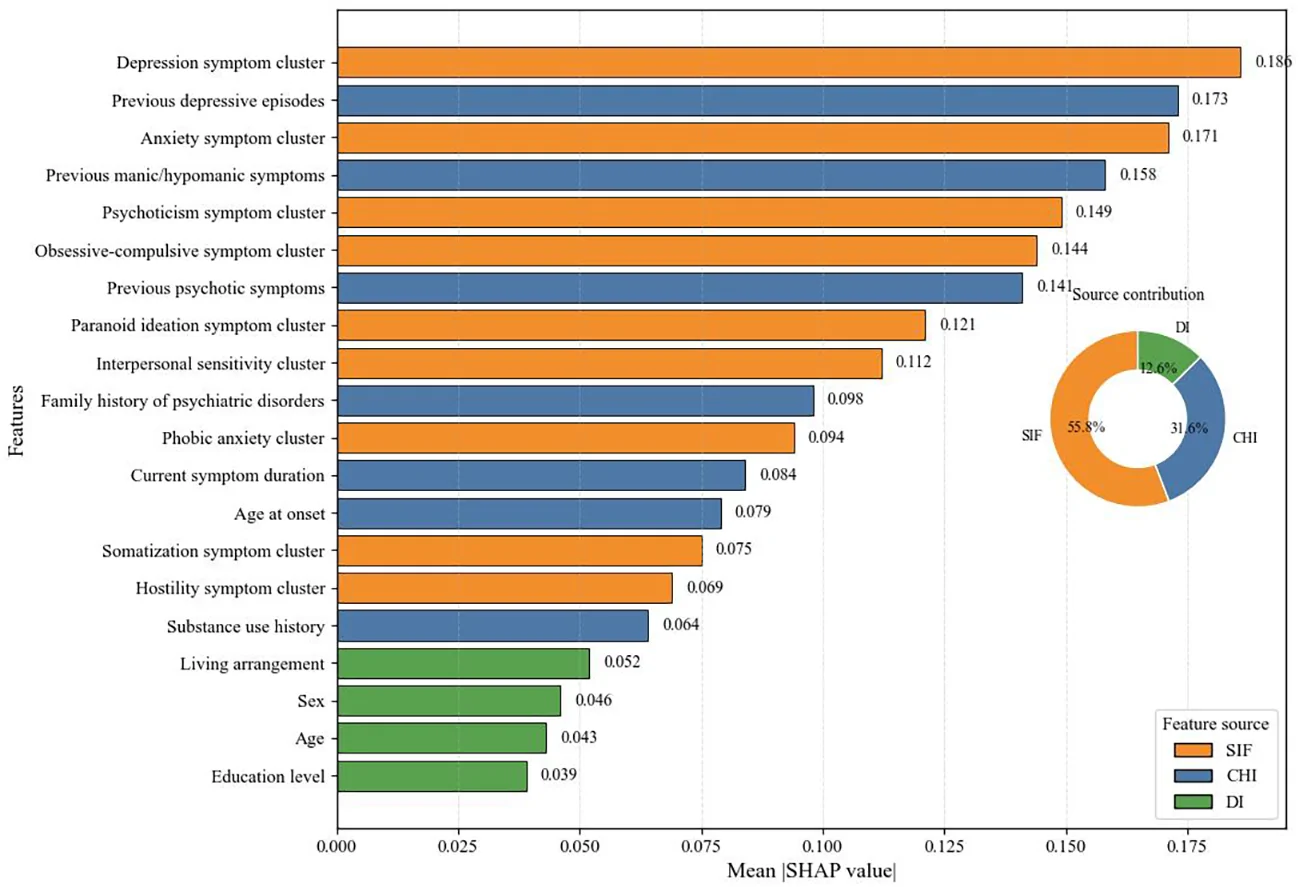
Macro-averaged absolute SHAP importance across the five diagnostic labels for predefined SCL-90 factors and multisource clinical features.

**Figure 11:**
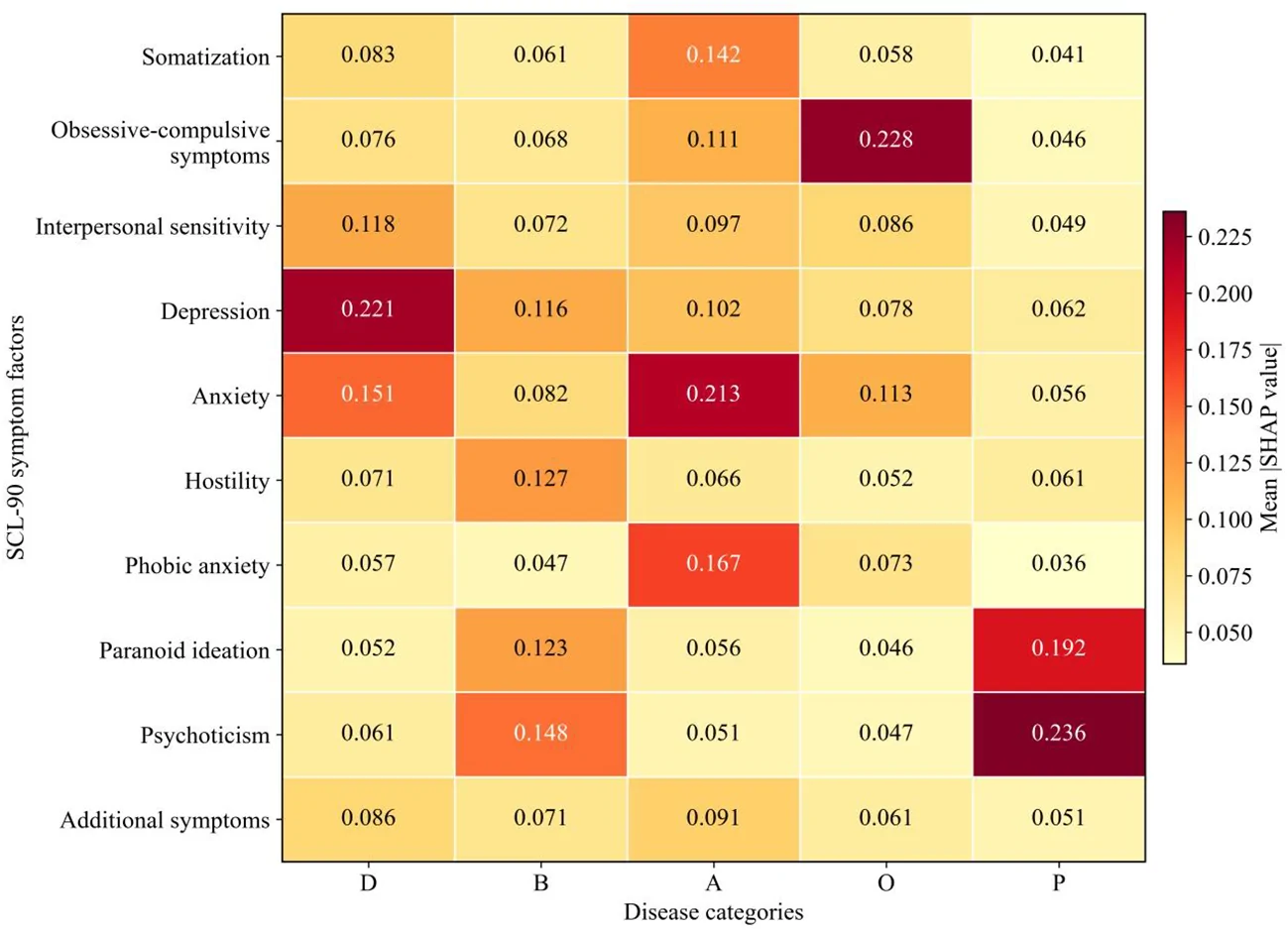
Disease-specific interpretation of SCL-90 symptom factors using SHAP analysis.

SHAP analysis was implemented using GradientExplainer for the TUCH-FT model trained with seed 42. A multilabel-stratified random sample of 200 training patients was used as the background dataset, and all 1272 patients in the temporal same-centre validation cohort were explained. SHAP values were calculated separately for each diagnostic label. Item-level absolute SHAP values within each predefined SCL-90 factor were averaged to obtain factor-level importance. **Figure 10** presents the macro-average across the five label-specific results, whereas **Figure 11** shows the attribution pattern for each label separately.

As shown in **Figure 10**, SCL-90 symptom features had the largest macro-averaged absolute SHAP values, followed by clinical history variables, whereas demographic variables had smaller attribution magnitudes. This pattern indicates that the model’s outputs were more sensitive to current symptom profiles and clinical history than to demographic characteristics.

**Figure 11** shows that the attribution patterns differed across diagnostic labels. Depressive and anxiety-related factors had relatively large attribution values across several outputs, whereas obsessive-compulsive symptoms, psychoticism and paranoid ideation showed more label-specific patterns. These SHAP results describe the predictive behaviour of TUCH-FT and should not be interpreted as causal effects, definitive diagnostic criteria or explanations of individual prediction errors.

### 3.7 Model ablation experiments

To evaluate the contribution and stability of each component of TUCH-FT, the FT-Transformer was used as the baseline, and TCFE, UE, CMAF and HMC were sequentially incorporated. Each configuration was independently trained and evaluated using five random seeds. **Table 7** reports the mean performance and standard deviation across the five runs, whereas **Table 8** presents paired bootstrap comparisons with the baseline FT-Transformer and between adjacent ablation configurations.

**Table 7:** Ablation performance of the TUCH-FT model across five independent runs.

Model	TCFE	UE	CMAF	HMC	Macro-F1↑	Micro-F1↑	Macro-AP↑	Hamming loss↓	LRAP↑
FT-Transformer					0.730 ± 0.006	0.775 ± 0.005	0.760 ± 0.007	0.128 ± 0.004	0.813 ± 0.006
FT + TCFE	✓				0.758 ± 0.005	0.786 ± 0.006	0.773 ± 0.006	0.122 ± 0.003	0.819 ± 0.005
FT + TCFE + UE	✓	✓			0.790 ± 0.004	0.818 ± 0.005	0.816 ± 0.005	0.106 ± 0.003	0.842 ± 0.004
FT + TCFE + UE + CMAF	✓	✓	✓		0.795 ± 0.005	0.837 ± 0.004	0.831 ± 0.004	0.102 ± 0.002	0.849 ± 0.004
TUCH-FT	✓	✓	✓	✓	0.803 ± 0.004	0.842 ± 0.004	0.838 ± 0.004	0.095 ± 0.002	0.876 ± 0.003

**Table 8:** Paired bootstrap comparisons of Macro-F1 among the TUCH-FT ablation configurations.

Comparison type	Comparison	ΔMacro-F1	Paired bootstrap 95% CI	Interpretation
Versus baseline	FT + TCFE vs. FT-Transformer	+0.028	[0.021, 0.035]	CI excludes zero
Versus baseline	FT + TCFE + UE vs. FT-Transformer	+0.060	[0.052, 0.068]	CI excludes zero
Versus baseline	FT + TCFE + UE + CMAF vs. FT-Transformer	+0.065	[0.057, 0.073]	CI excludes zero
Versus baseline	TUCH-FT vs. FT-Transformer	+0.073	[0.064, 0.082]	CI excludes zero
Incremental	FT + TCFE + UE vs. FT + TCFE	+0.032	[0.025, 0.039]	CI excludes zero
Incremental	FT + TCFE + UE + CMAF vs. FT + TCFE + UE	+0.005	[−0.001, 0.011]	CI includes zero
Incremental	TUCH-FT vs. FT + TCFE + UE + CMAF	+0.008	[0.002, 0.014]	CI excludes zero

ΔMacro-F1 represents the paired difference between the first and second models listed in each comparison. The 95% confidence intervals were estimated using 1000 paired bootstrap resamples of the test set, with identical resampled patients used for both models. Comparisons with the baseline evaluate cumulative improvements, whereas incremental comparisons evaluate the contribution of each newly added module. A difference was considered statistically supported when its 95% confidence interval did not include zero.

As shown in **Tables 7**, **8**, the addition of TCFE increased Macro-F1 from 0.730 ± 0.006 to 0.758 ± 0.005, with a paired improvement of 0.028 (95% CI: 0.021–0.035). UE further increased Macro-F1 to 0.790 ± 0.004, corresponding to an incremental improvement of 0.032 (95% CI: 0.025–0.039).

CMAF increased Macro-F1 numerically to 0.795 ± 0.005; however, its incremental difference of 0.005 had a 95% CI of −0.001–0.011, which included zero. Therefore, its isolated contribution to Macro-F1 should be interpreted cautiously. After incorporating HMC, TUCH-FT achieved the best overall performance, with a Macro-F1 of 0.803 ± 0.004, Micro-F1 of 0.842 ± 0.004, Macro-AP of 0.838 ± 0.004, Hamming Loss of 0.095 ± 0.002 and LRAP of 0.876 ± 0.003. The complete model improved Macro-F1 by 0.073 relative to the FT-Transformer (95% CI: 0.064–0.082).

Overall, the paired analyses support the contributions of TCFE, UE and HMC, whereas the smaller Macro-F1 improvement associated with CMAF remained within the range of sampling variation.

### 3.8 Visualisation of feature distributions

To further investigate how the proposed modules influence the discriminative characteristics of different mental disorders, we employed t-SNE to project the high-dimensional latent features extracted by the model at each ablation stage onto a two-dimensional space, as shown in **Figure 12**.

**Figure 12:**
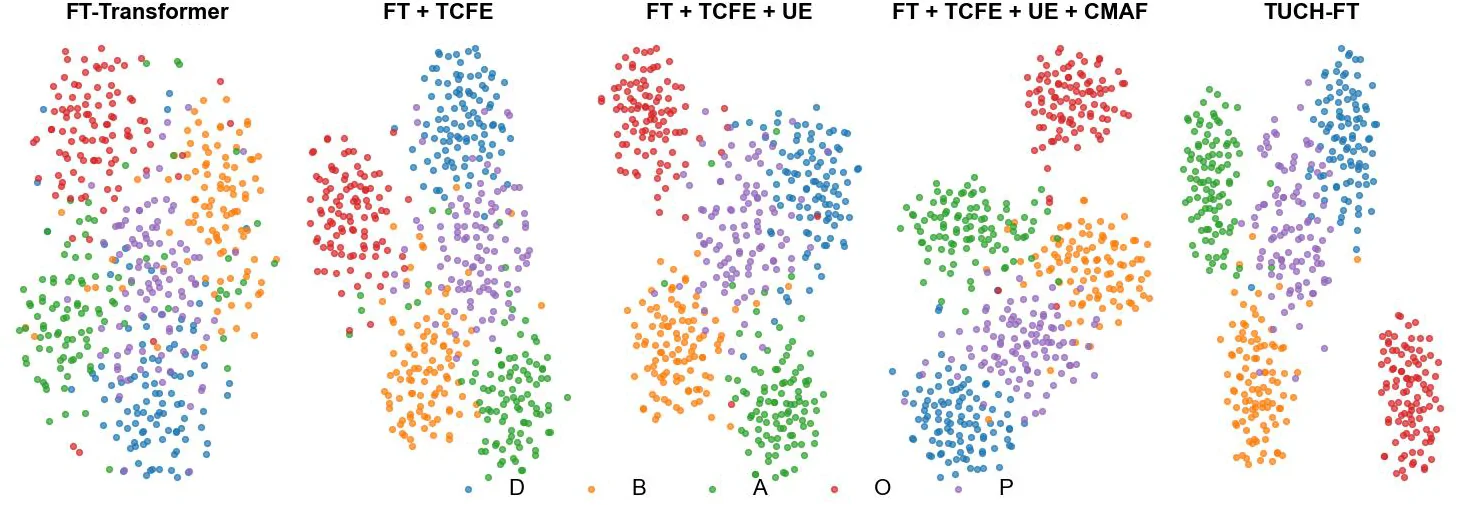
t-SNE visualization of latent feature distributions across different ablation stages.

The feature distributions generated by the original FT-Transformer exhibited significant overlap across the five disease categories, indicating limited inter-class separability. With the sequential integration of TCFE, UE and CMAF, class boundaries gradually became more distinct, whilst intra-class distributions became more compact. Compared with FT + TCFE + UE, the addition of CMAF was associated with a slight visual improvement in the separation of some diagnostic groups in **Figure 12**. This suggests a possible benefit for the learned feature representations, although its incremental Macro-F1 gain was not statistically supported (**Table 8**). Following the integration of HMC, TUCH-FT exhibited the most distinct clustering structure, with reduced overlap between different disease categories and enhanced intra-class compactness. These results indicate that the proposed modules progressively enhance the discriminative representation of multi-source clinical features, which is consistent with the improvements observed in the quantitative ablation results.

### 3.9 Clinical validation based on the TUCH-FT model

To further evaluate the reliability of the TUCH-FT model’s probabilistic predictions in clinical practice, a temporally separated cohort of 1272 patients was recruited from the same hospital between January 2024 and January 2025. These patients did not overlap with the model-development cohort and were not used for model training, model selection, hyperparameter tuning or decision-threshold determination. Therefore, this cohort was regarded as a temporal same-centre validation cohort rather than an independent clinical validation cohort. Using the fixed model and prespecified decision thresholds, calibration curves were plotted separately for depressive disorders, bipolar disorders, anxiety disorders, obsessive-compulsive disorders and psychotic disorders, and the models’ probabilistic calibration performance was quantitatively assessed using the Brier score and the expected calibration error (ECE).

The Brier score is used to measure the mean squared error between the predicted probability and the true label, the calculation method is shown in Equation 22:

(22) $$Brier=\frac{1}{N}\sum_{i=1}^{N}{\left(p_{i}-y_{i}\right)}^{2}$$

ECE is used to measure the difference between the average predicted probability and the actual positive rate within each predicted probability interval. The calculation method is shown in Equation 23:

(23) $$ECE=\sum_{m=1}^{M}\frac{\left|B_{m}\right|}{N}\left|acc\left(B_{m}\right)-conf\left(B_{m}\right)\right|$$

where, *N* denotes the number of samples, *p_i_* denotes the predicted probability of the *i*-th sample, and *y_i_* denotes the corresponding ground-truth label. *M* denotes the number of probability bins, *B_m_* denotes the set of samples within the *m*-th probability bin, acc(*B_m_*) denotes the actual positive rate for that bin, and conf(*B_m_*) represents the average predicted probability of samples within this interval.

Because the Brier score is influenced by label prevalence, a prevalence-based reference model assigning each patient the observed prevalence of label *c* was used. The reference Brier score and Brier skill score (BSS) were calculated as Equation 24, 25:

(24) $$BS_{\text{ref},c}=\pi_{c}\left(1-\pi_{c}\right)$$

(25) $$BSS_{c}=1-\frac{BS_{c}}{BS_{\text{ref},c}}$$

where πc is the prevalence of label *c*. A positive BSS indicates better probabilistic performance than the prevalence-based reference model.

The calibration curves are shown in **Figure 13**, and the Brier score, prevalence-based reference Brier score, BSS and ECE results are summarised in **Table 9**. The closer the calibration curve is to the ideal diagonal line, the more closely the model’s predicted probabilities align with the actual incidence of the disease; the lower the Brier score and ECE, the more reliable the model’s probability predictions are.

**Figure 13:**
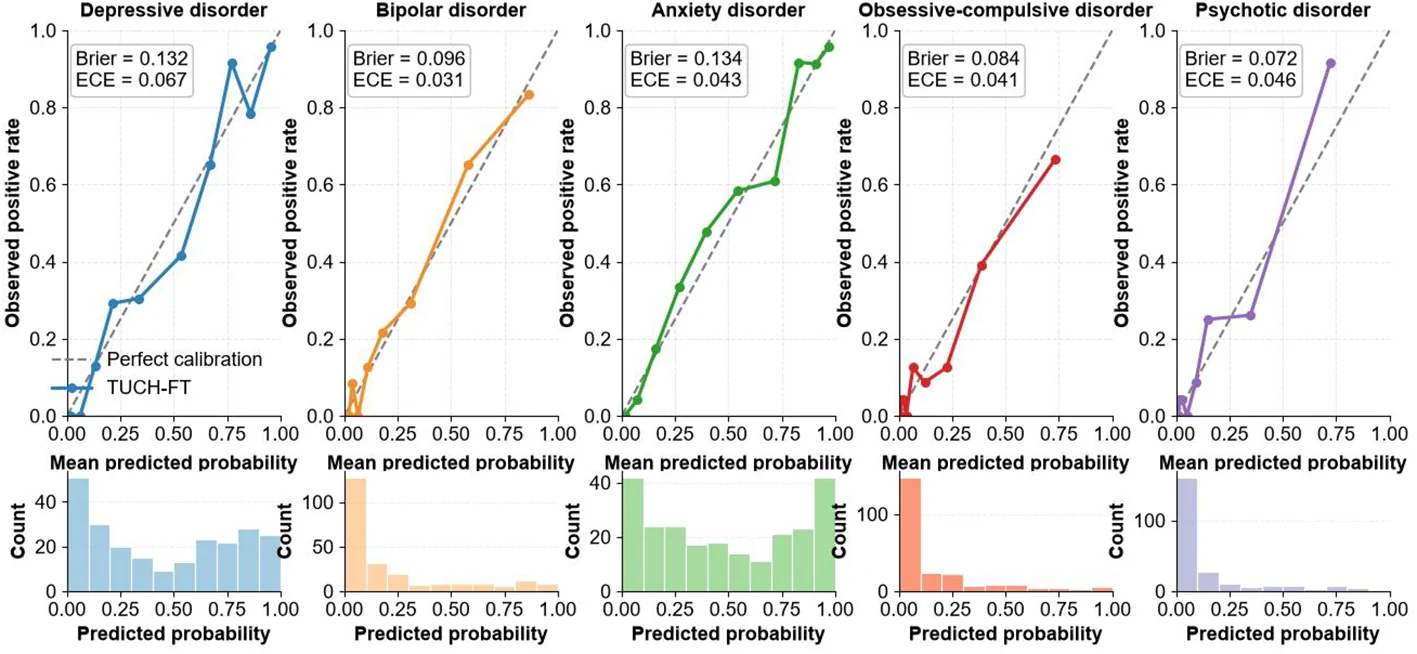
Probability calibration curves for different disease labels in the clinical validation of the TUCH-FT model.

**Table 9:** Calibration performance relative to the prevalence-based reference model in the temporal same-centre validation cohort.

Diagnostic label	Prevalence	TUCH-FT Brier score	Reference Brier score	BSS	ECE
Depressive disorder	0.510	0.132	0.250	0.472	0.067
Bipolar disorder	0.388	0.096	0.238	0.596	0.031
Anxiety disorder	0.556	0.134	0.247	0.457	0.043
Obsessive-compulsive disorder	0.396	0.084	0.239	0.649	0.041
Psychotic disorder	0.335	0.072	0.223	0.677	0.046

As shown in **Table 9**, the Brier scores ranged from 0.072 for psychotic disorder to 0.134 for anxiety disorder. Because raw Brier scores are influenced by label prevalence, the model scores were further compared with those of the prevalence-based reference model. TUCH-FT achieved positive BSS values for all five diagnostic labels, ranging from 0.457 to 0.677, indicating that its probabilistic predictions consistently outperformed the prevalence-only reference model. Psychotic disorder achieved a BSS of 0.677, suggesting that its relatively low Brier score was not solely attributable to its lower prevalence.

Bipolar disorder had the lowest ECE at 0.031, followed by obsessive-compulsive disorder, anxiety disorder and psychotic disorder at 0.041, 0.043 and 0.046, respectively. Depressive disorder had the highest ECE at 0.067, indicating relatively greater deviation between predicted and observed probabilities. Overall, calibration was interpreted jointly using the Brier score, BSS, ECE and calibration curves rather than the raw Brier score alone.

The predicted probability distributions further indicate that different disease labels exhibit distinct risk output characteristics. The predicted probability distributions for depressive disorders and anxiety disorders are relatively broad, suggesting that the model is capable of distinguishing between patients at different risk levels with a reasonable degree of precision; in contrast, the predicted probabilities for bipolar disorder, obsessive-compulsive disorder and psychotic disorders are more concentrated in the low-probability intervals, with only a proportion of patients receiving higher predicted probabilities, reflecting the model’s relative conservatism in identifying conditions with low prevalence. Overall, the TUCH-FT model is not only capable of producing disease classification results but also of providing individualised probability of disease with a high degree of reliability, thereby offering clinicians an auxiliary basis for disease risk stratification, differential diagnosis and targeted follow-up consultations.

## 4 Discussion

This study developed the TUCH-FT model for patients attending their first psychiatric consultation; this model is capable of providing preliminary auxiliary diagnoses by simultaneously utilising item-level information from the DI, CHI and SIF scales. Its primary advantages lie in the model’s ability to preserve the structural characteristics of different clinical information sources, identify the potential diagnostic value within ‘unclear’ medical histories, and learn the interactive relationships between patient background, past medical history and current symptoms. Furthermore, the hierarchical multi-label output better aligns with the clinical characteristics of psychiatric comorbidity and diagnostic overlap.

Although TUCH-FT achieved a 60.8% exact-match accuracy, identifying each patient’s complete diagnostic profile remains challenging. This study still has certain limitations. Firstly, both the model development and temporal same-centre validation cohorts were obtained from a single hospital in Harbin, China. Therefore, the study population may be relatively homogeneous in terms of geographic background, ethnicity, socioeconomic characteristics and healthcare-seeking behaviour. Moreover, psychiatric diagnostic thresholds and the clinical interpretation of overlapping symptoms may vary across regions, healthcare institutions and cultural settings. These demographic and clinical differences may affect the model’s performance and limit its generalisability to other populations. In addition, restricting enrolment to patients aged 18–55 years with mood-related symptoms may have under-represented the broader spectrum of psychotic and obsessive-compulsive disorders. Although the AUC for psychotic disorder was 0.906, this result reflects discrimination within the recruited cohort and does not establish equivalent performance among patients presenting primarily with psychotic symptoms.

Secondly, clinical histories and SCL-90 results may still be subject to recall bias, insufficient insight and differences in understanding; whilst some ‘unclear’ information is retained by the model, its true clinical significance still requires verification in conjunction with more objective data.

Thirdly, the temporal validation cohort had a higher proportion of patients with the target diagnoses, indicating a difference in case mix between recruitment periods. This allowed assessment of model performance in a cohort with higher diagnostic prevalence, although the factors underlying the shift were not formally evaluated. The applicability of the fixed thresholds and calibration results to other patient populations therefore requires further validation. Direct comparison with published models also remains unavailable because the studies used different datasets and prediction tasks.

Future research should expand the sample size, conduct multi-centre prospective external validation, and further integrate electronic health records, clinician assessments and follow-up data to enhance the model’s stability, interpretability and clinical applicability.

## 5 Conclusions

This study consecutively enrolled patients attending a psychiatric clinic for the first time, collected item-level scores for the DI, CHI and SIF, and determined the final diagnostic labels through longitudinal follow-up, treatment response and comprehensive consultation with multiple psychiatric specialists. On this basis, the TUCH-FT multi-label prediction model, which integrates TCFE, UE, CMAF and HMC, was developed.

The results demonstrated that TUCH-FT achieved Macro-F1, Micro-F1, Macro-AP, Hamming Loss and LRAP values of 0.803, 0.842, 0.838, 0.095 and 0.876, respectively. Compared with FT-Transformer, Macro-F1, Micro-F1, Macro-AP and LRAP improved by 10.0%, 8.6%, 10.3% and 7.7% respectively, whilst Hamming Loss was reduced by 25.8%. In clinical validation involving 1272 independent new patients, the Brier scores for the five disease categories ranged from 0.072 to 0.134, with ECE values ranging from 0.031 to 0.067, indicating favourable classification and calibration performance in this temporal same-centre validation cohort.

In summary, TUCH-FT is capable of integrating patients’ baseline characteristics, medical history and current symptoms to provide an auxiliary basis for disease screening, risk stratification, comorbidity identification and differential diagnosis in patients attending a psychiatric clinic for the first time.

Future work will evaluate TUCH-FT in prospective multi-centre cohorts and examine whether integrating electronic health records and longitudinal clinical data improves its generalisability and clinical utility.

## Data Availability

The raw data supporting the conclusions of this article will be made available by the authors, without undue reservation.
