# Supplementary material for: A multisource clinical deep learning model for early identification of mental disorders in first-visit psychiatric patients
Source: Front Psychiatry. 2026 Oct 28;17:1988768. doi: 10.3389/fpsyt.2026.1988768 (PMC13752122; doi:10.3389/fpsyt.2026.1988768)
Supplement: Supplementary file 2 [file Supplementaryfile1.docx]

Supplementary Material

# 1 Supplementary Figures and Tables

For more information on Supplementary Material and for details on the different file types accepted, please see [here](https://www.frontiersin.org/guidelines/author-guidelines#supplementary-material).

## 1.1 Supplementary Figures

Figure 1. The severity levels of symptoms corresponding to each score.

Figure 2. The distribution of factor mean scores among the enrolled patients.

Figure 3. Overall structure of the TUCH-FT model.

Figure 4. Schematic diagram of the TCFE method.

Figure 5. Architecture of the uncertainty-aware embedding (UE) module.

Figure 6. Principles of the CMAF mechanism and a cross-group fusion structure of three sets of clinical features.

Figure 7. Schematic diagram of the HMC structure.

|  |  |
| --- | --- |
| (a) Ground-truth comorbidity patterns | (b) TUCH-FT predicted comorbidity patterns |

Figure 8. Comorbidity pattern analysis of TUCH-FT predictions.

Figure 9. Combination-level confusion matrix between clinical diagnoses and model predictions

Figure 10. Macro-averaged absolute SHAP importance across the five diagnostic labels for predefined SCL-90 factors and multisource clinical features

Figure 11. Disease-specific interpretation of SCL-90 symptom factors using SHAP analysis

Figure 12. t-SNE visualization of latent feature distributions across different ablation stages

Figure 13. Probability calibration curves for different disease labels in the clinical validation of the TUCH-FT model.

## 1.2 Supplementary Tables

Table 1. Prevalence of the target diagnostic labels in the model-development cohort.

| Diagnostic label | Positive cases | Prevalence |
| --- | --- | --- |
| Depression | 1655 | 28.5% |
| Bipolar disorder | 1260 | 21.7% |
| Anxiety disorder | 1803 | 31.1% |
| Obsessive-compulsive disorder | 1285 | 22.1% |
| Psychotic disorder | 1086 | 18.7% |
| At least one positive target label | 3243 | 55.9% |
| No positive target label | 2559 | 44.1% |

Note: The diagnostic labels are not mutually exclusive, as a single patient may have multiple diagnosed mental disorders. Consequently, the sum of the patient counts and percentages across these labels exceeds the total number of patients or 100%.

Table 2. Baseline DI, CHI, and SCL-90 characteristics of first-visit psychiatric patients.

| **Category** | **Variable** | **Clinical relevance** | **Category** | **n (%) or mean ± SD** |
| --- | --- | --- | --- | --- |
| DI | age | reflects disease risk differences across age groups | Overall | 34.6 ± 10.2 |
|  |  |  | 18-27 | 1857 (32.0%) |
|  |  |  | 28-36 | 1683 (29.0%) |
|  |  |  | 37-45 | 870 (15.0%) |
|  |  |  | 46-55 | 1392 (24.0%) |
|  | sex | evaluates differences in disease distribution between sexes | Male | 2205 (38.0%) |
|  |  |  | Female | 3597 (62.0%) |
|  | education level | evaluates the influence of education level on SCL-90 comprehension and responses | Primary school | 696 (12.0%) |
|  |  |  | Junior high school | 1102 (19.0%) |
|  |  |  | Senior high school | 1335 (23.0%) |
|  |  |  | College or above | 2669 (46.0%) |
|  | marital status | reflects social and family support conditions | Single | 928 (16.0%) |
|  |  |  | Married | 2611 (45.0%) |
|  |  |  | Divorced | 1857 (32.0%) |
|  |  |  | Widowed | 406 (7.0%) |
|  | employment status | evaluates social functioning and living status | Employed | 2785 (48.0%) |
|  |  |  | Student | 1683 (29.0%) |
|  |  |  | Unemployed | 1044 (18.0%) |
|  |  |  | Retired | 290 (5.0%) |
|  | living arrangement | reflects daily support and caregiving conditions | Living alone | 3075 (53.0%) |
|  |  |  | Living with family | 2727 (47.0%) |
| CHI | age at onset | evaluates early- or late-onset disease characteristics | N/A | 26.8 ± 7.4 |
|  | current symptom duration | reflects the duration of the current episode | N/A | 8.6 ± 5.7 |
|  | family history of psychiatric disorders | evaluates genetic and familial risk factors | Yes | 1741 (30.0%) |
|  |  |  | No | 3249 (56.0%) |
|  |  |  | Unclear | 812 (14.0%) |
|  | substance use history | identifies or excludes substance-related psychiatric symptoms | Yes | 1160 (20.0%) |
|  |  |  | No | 4177 (72.0%) |
|  |  |  | Unclear | 465 (8.0%) |
|  | previous suspected psychotic symptoms | assists in identifying schizophrenia spectrum disorders | Yes | 1160 (20.0%) |
|  |  |  | No | 3945 (68.0%) |
|  |  |  | Unclear | 697 (12.0%) |
|  | previous suspected manic or hypomanic symptoms | assists in identifying bipolar disorders | Yes | 2205 (38.0%) |
|  |  |  | No | 2553 (44.0%) |
|  |  |  | Unclear | 1044 (18.0%) |
|  | previous suspected depressive episodes | assists in identifying depressive disorders | Yes | 3713 (64.0%) |
|  |  |  | No | 1160 (20.0%) |
|  |  |  | Unclear | 929 (16.0%) |
|  | previous significant anxiety or panic symptoms | assists in identifying anxiety disorders | Yes | 3017 (52.0%) |
|  |  |  | No | 1973 (34.0%) |
|  |  |  | Unclear | 812 (14.0%) |
|  | previous dissociative or conversion symptoms | assists in identifying dissociative and conversion disorders | Yes | 1276 (22.0%) |
|  |  |  | No | 3597 (62.0%) |
|  |  |  | Unclear | 929 (16.0%) |
|  | previous obsessive-compulsive symptoms | assists in identifying obsessive-compulsive disorder | Yes | 1973 (34.0%) |
|  |  |  | No | 2785 (48.0%) |
|  |  |  | Unclear | 1044 (18.0%) |

Table 3. Composition and assessment content of the SCL-90 Factors.

| **Factor** | **Number of Items** | **Main Assessment Domain** | **Typical Manifestations** |
| --- | --- | --- | --- |
| Somatization | 12 | physical discomfort and somatic manifestations of anxiety | headache, chest pain, dizziness, gastrointestinal discomfort, muscle pain |
| obsessive-compulsive symptoms | 10 | uncontrollable thoughts, impulses, or behaviors | Repeated checking, repetitive hand washing, persistent meaningless thoughts |
| interpersonal sensitivity | 9 | feelings of discomfort, inferiority, and self-consciousness in interpersonal interactions | Feeling inferior to others, being easily hurt, feeling misunderstood |
| depression | 13 | depressive mood and related cognitive and behavioral symptoms | Depressed mood, loss of interest, feelings of worthlessness, hopelessness |
| anxiety | 10 | tension, irritability, panic, and associated physical responses | Palpitations, trembling, restlessness, sudden fear |
| hostility | 6 | hostile emotions, irritability, and aggressive impulses | Easily losing temper, arguments, throwing objects, thoughts of harming others |
| phobic anxiety | 7 | excessive fear of specific places or situations | Fear of going out alone, fear of crowds, fear of transportation |
| paranoid ideation | 6 | suspicion, hostility, and persecutory experiences | Feeling monitored, distrust of others, believing others intend to take advantage |
| psychoticism | 10 | abnormal perception, thought disturbances, and social detachment experiences | Hearing voices that others cannot hear, feeling controlled by thoughts |
| additional symptoms | 7 | sleep, appetite, and other miscellaneous psychological problems | Difficulty falling asleep, early awakening, poor sleep quality, appetite changes |

Table 4. A Comparison of the performance of different models in multi-label classification tasks for mental disorders.

| **Model** | **Model type** | **Macro-F1↑** | **Micro-F1↑** | **Macro-AP↑** | **Hamming Loss↓** | **LRAP↑** |
| --- | --- | --- | --- | --- | --- | --- |
| LR | linear classification models | 0.540 | 0.612 | 0.575 | 0.236 | 0.642 |
| RF | ensemble tree models | 0.578 | 0.648 | 0.612 | 0.214 | 0.671 |
| XGBoost | gradient-boosted tree models | 0.612 | 0.681 | 0.645 | 0.193 | 0.703 |
| MLP | basic deep learning models | 0.668 | 0.724 | 0.701 | 0.162 | 0.748 |
| FT-Transformer | table models | 0.730 | 0.775 | 0.760 | 0.128 | 0.813 |
| TUCH-FT | the improved model proposed in this paper | 0.803 | 0.842 | 0.838 | 0.095 | 0.876 |

Notes: “↑” indicates that higher values correspond to better performance_1_; “↓” indicates that lower values correspond to better performance_2_.

Table 5. Label-specific discrimination performance of TUCH-FT in the temporal same-centre validation cohort.

| **Diagnostic label** | **Positive (%)** | **Precision** | **Recall** | **F1 score** | **AUC** | **AP** |
| --- | --- | --- | --- | --- | --- | --- |
| Depression disorder | 649 (51.0%) | 0.838 | 0.812 | 0.825 | 0.902 | 0.854 |
| Bipolar disorder | 494 (38.8%) | 0.792 | 0.758 | 0.775 | 0.887 | 0.817 |
| Anxiety disorder | 707 (55.6%) | 0.844 | 0.821 | 0.832 | 0.915 | 0.872 |
| Obsessive-compulsive disorder | 504 (39.6%) | 0.806 | 0.772 | 0.789 | 0.891 | 0.824 |
| Psychotic disorder | 426 (33.5%) | 0.817 | 0.774 | 0.795 | 0.906 | 0.823 |
| Macro-average | N/A | 0.819 | 0.787 | 0.803 | 0.900 | 0.838 |

Table 6. Results of experiments with different combinations of input features.

| **Features** | | | **Macro-F1↑** | **Micro-F1↑** | **Macro-AP↑** | **HammingLoss↓** | **LRAP↑** |
| --- | --- | --- | --- | --- | --- | --- | --- |
| DI | CHI | SIF |  |  |  |  |  |
| √ |  |  | 0.126 | 0.193 | 0.154 | 0.342 | 0.287 |
|  | √ |  | 0.438 | 0.482 | 0.451 | 0.246 | 0.536 |
|  |  | √ | 0.451 | 0.497 | 0.466 | 0.239 | 0.548 |
| √ | √ |  | 0.701 | 0.742 | 0.719 | 0.146 | 0.781 |
| √ |  | √ | 0.728 | 0.768 | 0.747 | 0.134 | 0.806 |
|  | √ | √ | 0.756 | 0.791 | 0.778 | 0.121 | 0.829 |
| √ | √ | √ | 0.803 | 0.842 | 0.838 | 0.095 | 0.876 |

Table 7. Ablation performance of the TUCH-FT model across five independent runs.

| **Model** | **TCFE** | **UE** | **CMAF** | **HMC** | **Macro-F1↑** | **Micro-F1↑** | **Macro-AP↑** | **Hamming Loss↓** | **LRAP↑** |
| --- | --- | --- | --- | --- | --- | --- | --- | --- | --- |
| FT-Transformer |  |  |  |  | 0.730 ± 0.006 | 0.775 ± 0.005 | 0.760 ± 0.007 | 0.128 ± 0.004 | 0.813 ± 0.006 |
| FT + TCFE | √ |  |  |  | 0.758 ± 0.005 | 0.786 ± 0.006 | 0.773 ± 0.006 | 0.122 ± 0.003 | 0.819 ± 0.005 |
| FT + TCFE + UE | √ | √ |  |  | 0.790 ± 0.004 | 0.818 ± 0.005 | 0.816 ± 0.005 | 0.106 ± 0.003 | 0.842 ± 0.004 |
| FT + TCFE + UE + CMAF | √ | √ | √ |  | 0.795 ± 0.005 | 0.837 ± 0.004 | 0.831 ± 0.004 | 0.102 ± 0.002 | 0.849 ± 0.004 |
| TUCH-FT | √ | √ | √ | √ | 0.803 ± 0.004 | 0.842 ± 0.004 | 0.838 ± 0.004 | 0.095 ± 0.002 | 0.876 ± 0.003 |

Table 8 Paired bootstrap comparisons of Macro-F1 among the TUCH-FT ablation configurations.

| **Comparison type** | **Comparison** | **ΔMacro-F1** | **Paired bootstrap 95% CI** | **Interpretation** |
| --- | --- | --- | --- | --- |
| Versus baseline | FT + TCFE vs. FT-Transformer | +0.028 | [0.021, 0.035] | CI excludes zero |
| Versus baseline | FT + TCFE + UE vs. FT-Transformer | +0.060 | [0.052, 0.068] | CI excludes zero |
| Versus baseline | FT + TCFE + UE + CMAF vs. FT-Transformer | +0.065 | [0.057, 0.073] | CI excludes zero |
| Versus baseline | TUCH-FT vs. FT-Transformer | +0.073 | [0.064, 0.082] | CI excludes zero |
| Incremental | FT + TCFE + UE vs. FT + TCFE | +0.032 | [0.025, 0.039] | CI excludes zero |
| Incremental | FT + TCFE + UE + CMAF vs. FT + TCFE + UE | +0.005 | [−0.001, 0.011] | CI includes zero |
| Incremental | TUCH-FT vs. FT + TCFE + UE + CMAF | +0.008 | [0.002, 0.014] | CI excludes zero |

Table 9. Calibration performance relative to the prevalence-based reference model in the temporal same-centre validation cohort.

| **Diagnostic label** | **Prevalence** | **TUCH-FT Brier score** | **Reference Brier score** | **BSS** | **ECE** |
| --- | --- | --- | --- | --- | --- |
| Depressive disorder | 0.510 | 0.132 | 0.250 | 0.472 | 0.067 |
| Bipolar disorder | 0.388 | 0.096 | 0.238 | 0.596 | 0.031 |
| Anxiety disorder | 0.556 | 0.134 | 0.247 | 0.457 | 0.043 |
| Obsessive-compulsive disorder | 0.396 | 0.084 | 0.239 | 0.649 | 0.041 |
| Psychotic disorder | 0.335 | 0.072 | 0.223 | 0.677 | 0.046 |
